# The Yeast Pif1 Helicase Prevents Genomic Instability Caused by G-Quadruplex-Forming CEB1 Sequences In Vivo

**DOI:** 10.1371/journal.pgen.1000475

**Published:** 2009-05-08

**Authors:** Cyril Ribeyre, Judith Lopes, Jean-Baptiste Boulé, Aurèle Piazza, Aurore Guédin, Virginia A. Zakian, Jean-Louis Mergny, Alain Nicolas

**Affiliations:** 1 Recombinaison et Instabilité Génétique, Institut Curie Centre de Recherche, CNRS UMR3244, Université Pierre et Marie Curie, Paris, France; 2 Department of Molecular Biology, Princeton University, Princeton, New Jersey, United States of America; 3 Laboratoire de Biophysique, Museum National d'Histoire Naturelle USM 503, INSERM U565, CNRS UMR5153, Paris, France; National Institute of Diabetes and Digestive and Kidney Diseases, United States of America

## Abstract

In budding yeast, the Pif1 DNA helicase is involved in the maintenance of both nuclear and mitochondrial genomes, but its role in these processes is still poorly understood. Here, we provide evidence for a new Pif1 function by demonstrating that its absence promotes genetic instability of alleles of the G-rich human minisatellite CEB1 inserted in the *Saccharomyces cerevisiae* genome, but not of other tandem repeats. Inactivation of other DNA helicases, including Sgs1, had no effect on CEB1 stability. In vitro, we show that CEB1 repeats formed stable G-quadruplex (G4) secondary structures and the Pif1 protein unwinds these structures more efficiently than regular B-DNA. Finally, synthetic CEB1 arrays in which we mutated the potential G4-forming sequences were no longer destabilized in *pif1Δ* cells. Hence, we conclude that CEB1 instability in *pif1Δ* cells depends on the potential to form G-quadruplex structures, suggesting that Pif1 could play a role in the metabolism of G4-forming sequences.

## Author Summary

Changes in the primary DNA sequence are a major source of pathologies and cancers. The hereditary information also resides in secondary DNA structures, a layer of genetic information that remains poorly understood. Biophysical and structural studies have long established that, in vitro, the DNA molecule can adopt diverse structures different from the canonical Watson-Crick conformations. However, for a long time their existence in vivo has been regarded with a certain skepticism and their functional role elusive. One example is the G-quadruplex structure, which involves G-quartets that form between four DNA strands. Here, using in vitro and in vivo assays in the yeast *S. cerevisiae*, we reveal the unexpected role of the Pif1 helicase in maintaining the stability of the human CEB1 G-rich tandem repeat array. By site-directed mutagenesis, we show that the genomic instability of CEB1 repeats in absence of Pif1 and is directly dependent on the ability of CEB1 to form G-quadruplex structures. We show that Pif1 is very efficient in vitro in processing G-quadruplex structures formed by CEB1. We propose that Pif1 maintains CEB1 repeats by its ability to resolve G-quadruplex structures, thus providing circumstantial evidence of their formation in vivo.

## Introduction

At the chromosomal level, in addition to coding regions and epigenetic modifications, the biological information also resides in DNA secondary structures, but this layer remains to be further deciphered. Biophysical and structural studies have long established that in vitro DNA can adopt diverse structures different from the canonical Watson-Crick conformations [1]. However, for a long time, the hypothesis that these structures occur in the native chromosomal context, as an integral part of the functional architecture of a chromosome, has been regarded with a certain skepticism. One example of such a non canonical DNA structure is the G-quadruplex, also named G-tetraplex or G4 DNA. These structures form in vitro in guanine-rich sequences that contain four tracts of at least three guanines separated by other bases, and are stabilized by G-quartets that form between four DNA strands [2]. Under physiological conditions, long runs of G4-forming sequences promote the formation of highly stable structures that can form spontaneously in vitro and, once formed, are very resistant to thermal denaturation. It is also important to consider that sequences that form G4-DNA slowly in vitro may be more prone to fold in vivo owing to the action of proteins that promote and/or stabilize their formation, such as the beta subunit of the ciliate *Oxytricha* telomere binding protein complex [3],[4].

Evidence for in vivo formation of G4 DNA has emerged in recent years. Notably, G4 DNA has been observed by electron microscopy from transcribed human G-rich DNA arrays in bacteria [5] and has been detected at the end of the ciliate *Oxytricha* telomeres by immunochemistry [6],[7]. As a complementary approach, genome-wide bioinformatic analyses have identified regions that have the potential to form G4 DNA within evolutionary diverse model systems, from bacteria to human. For example, in the human genome, more than 300,000 distinct sites have the potential to form G4 DNA [8],[9]. These sequences are highly over-represented in the promoter regions of diverse organisms, including human [10], yeast [11] and bacteria [12]. In addition, potential G4-forming sequences are found in G-rich arrays such as telomeres, rDNA or G-rich micro- and minisatellites. Hence, it has been suggested that their presence might affect transcriptional or post-transcriptional events when the G4 forming sequence is within the transcribed region [11],[13]. G4 DNA has also been proposed to participate in telomere capping, DNA replication and recombination [14]. However, it remains to be determined how and to what extent these secondary structures affect these processes and how they are maintained through DNA replication despite causing a structural impairment to the various nucleic acid processing enzymes.

It is clear that DNA goes through a single strand configuration locally during processes like DNA replication, transcription or repair, and many models argue that this single stranded stage favors G4 DNA formation [14]. In vitro, several DNA helicases, such as the human BLM, WRN, FANCJ and the *S. cerevisiae* Sgs1, can unwind G4 structures. They preferentially unwind G4 DNA over partially duplex DNA, forked DNA or Holliday junction substrates, and their helicase activity is inhibited in presence of G4 DNA ligands [15]–[18]. In *Caenorhabditis elegans* the FANCJ homolog *dog-1* is involved in the maintenance of G-rich regions by preventing intrinsic instability and loss of these regions [19],[20]. However, considering that different G-rich sequences can adopt very diverse secondary structures, and that in numerous instances genes encoding helicases are not essential, the questions of how many and which class of helicases are indeed able to process efficiently these secondary structures formed in guanine-rich regions in a given organism remains to be addressed. Also, until now, very few in vivo systems exist to study the involvement of helicases in processing these structures and assay artificially designed variant substrates.

In the present study, which was aimed at characterizing the mechanism(s) of rearrangement of tandem DNA repeats, we uncover an unexpected function of the Pif1 helicase with regards to processing G4 structures. Pif1 is a member of a conserved family of 5′-3′ DNA helicases, with distant homology to the RecD bacterial helicase. The *S. cerevisiae* Pif1 protein is important both for maintenance of mitochondrial DNA [21],[22] and as a negative regulator of telomerase-mediated telomere lengthening [23],[24]. Here we report that Pif1 also affects stability of the G-rich CEB1 minisatellite when it is inserted into a yeast chromosome. In contrast, mutations in other helicases, including the *S. cerevisiae* RecQ homologue Sgs1, had no effect on CEB1 stability. In vitro, CEB1 formed G4 structures that were efficiently unwound by Pif1. Finally, mutation of the CEB1 repeats such that they were no longer able to form G4 structures made them insensitive to Pif1. Thus we demonstrated that one of the functions of the Pif1 helicase is to process G4 structures. As sequences with the ability to form G4 DNA are found throughout the yeast genome, beyond acting on intrinsically instable repeats, we propose that the processing of G4 structures by Pif1 may facilitate DNA replication, transcription and/or repair.

## Results

### The DNA Helicase Pif1 Actively Destabilizes CEB1 during Vegetative Growth

**Figure 1:**
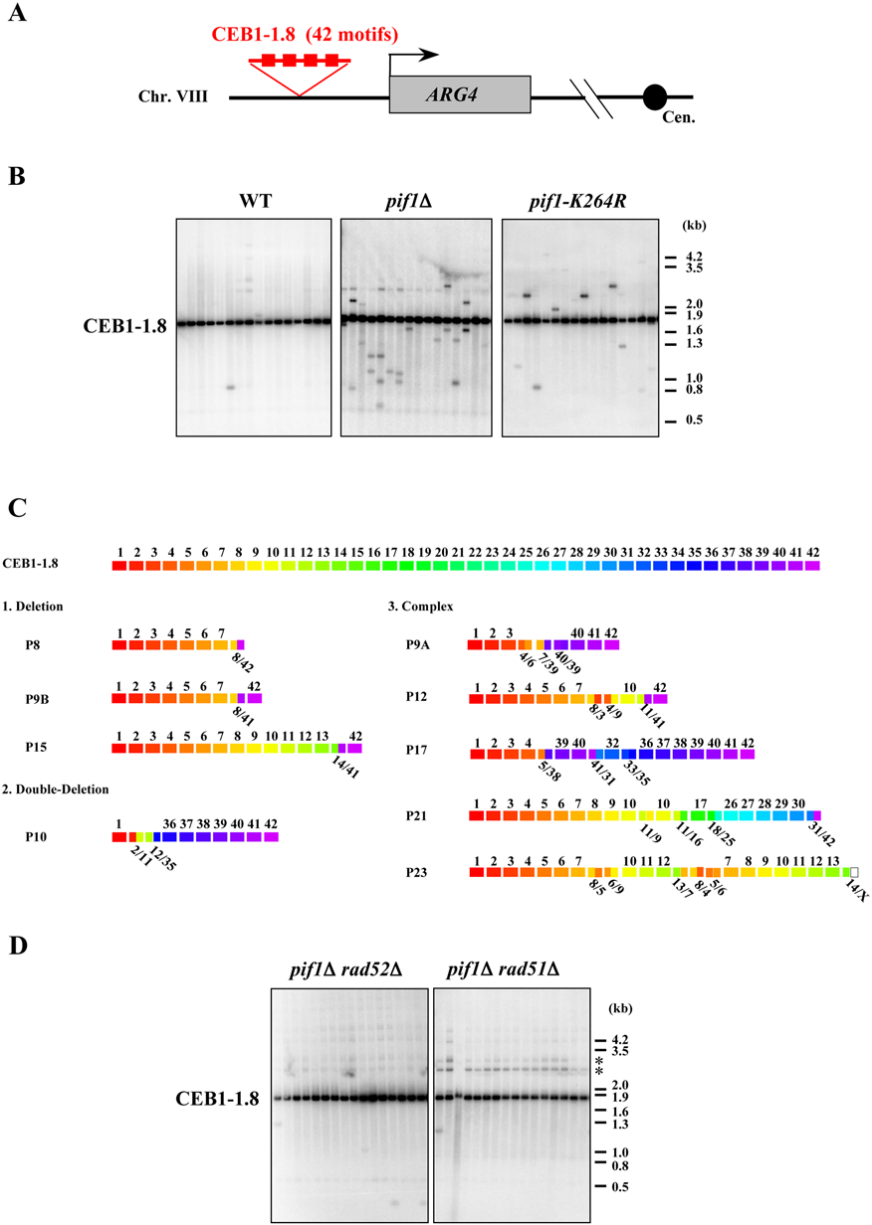
The CEB1 minisatellite is unstable in *pif1Δ* deficient cells. (A) Structure of the genomic locus containing CEB1-1.8. (B) Southern blot analysis of CEB1-1.8 instability in haploid strains: WT (ORT2914), *pif1Δ* (ORT4843), *pif1-K264R* (ORT5083-4E). Each lane contains DNA extracted from pools of 12 independent colonies digested by *Alu*I and hybridized with a CEB1-0.6 probe. (C) Structure of CEB1-1.8 rearrangements obtained in *pif1Δ* haploids. Each of the 42 CEB1-1.8 repeats is represented by a colored box and numbered (top). Nine rearrangements were sequenced and classified in three categories (1 to 3). The name of each rearranged allele is at the left. Hybrid repeats are represented by the two colors corresponding to the fused repeats. The white box in P23 indicates a motif that cannot be attributed to a specific parental motif. (D) CEB1 instability is Rad52 and Rad51 dependent. Southern blot analysis of CEB1-1.8 instability in *pif1Δ rad52Δ* (ORD7565-2C) and *pif1Δ rad51Δ* (ORD7574-9B) haploid strains. Same legends as in (B). Additional bands marked with an asterisk are presumably due to partial digestion by *Alu*I.

We previously developed yeast strains to study the genetic instability of a natural 1.8 kb allele of the human minisatellite CEB1 inserted in the S. *cerevisiae* genome (Figure 1A). This allele (called CEB1-1.8) is composed of a tandem array of 42 polymorphic repeats of sizes varying between 36 and 43 base pairs (bp) [25] (Figure S1). In our standard assay, which measures the frequency of allele size variation after growth for seven generations at 30°C, approximately 0.3% of wild-type (WT) cells exhibit a change in CEB1 size (contractions and expansions). Using this system, we reported that CEB1-1.8 was strongly destabilized in the absence of the Rad27/FEN1 endonuclease (42% instability) [26].

Recently, it was reported that the lethality caused by inactivation of the essential helicase/endonuclease Dna2, which participates with Rad27 in the maturation of Okazaki fragments, could be rescued by inactivation of the DNA helicase Pif1 [27]. These results prompted us to test if Pif1 also had an effect on the maintenance of CEB1 arrays in our system. Remarkably, in the absence of Pif1 (*pif1Δ*), the frequency of rearrangement by contractions or expansions of the parental allele increased 20-fold compared to WT cells (6% instability; Table 1, Figure 1B). As a control, a *pif1Δ* CEB1-1.8 strain containing a multicopy plasmid that expressed the WT *PIF1* gene under the control of the *PIF1* promoter did not exhibit CEB1 instability. Together, these results demonstrate that the absence of Pif1 destabilizes the CEB1-1.8 minisatellite at a rate of ∼1% per cell per generation. CEB1 instability was not specific to tracts inserted at the *ARG4* locus as CEB1-1.8 inserted at the *ADP1* locus in chromosome III was stable in the presence of Pif1 but was rearranged in its absence (3.6% instability; 7/192). The difference in stability between the two chromosomal locations is not statistically significant (Fisher's Exact test, p = 0.28).

**Table 1:** Instability of CEB1-1.8 in haploid strains.

Strain	Genotype	Number of rearrangements/total (%)	Fold increase vs. WT	p value vs. WT*
ORT2914	WT	5/1824 (0.3)	1	-
ORT4841	*pif1Δ*	40/672 (6.0)	20	<0.01
ORT4843	*pif1Δ*	11/192 (5.8)	19	<0.01
ORD7569	*pif1Δ/pif1Δ*	12/192 (6.3)	21	<0.01
ORT5083-4E	*pif1-K264R*	18/576 (3.2)	10	<0.01
ORT5087-5E	*pif1-K264A*	12/384 (3.2)	10	<0.01
ORT5084-2C	*pif1-m1*	1/192 (0.5)	2	NS
ORT5085-1C	*pif1-m2*	1/384 (0.3)	1	NS
ORT4848	*pif1Δ dna2Δ*	18/384 (4.7)	16	<0.01
ORT4880	*rrm3Δ*	0/336 (0)	<1	NS
ORD9304-9A	*pif1Δ rrm3Δ*	15/384 (3.9)	13	<0.01
ORT4849	*sgs1Δ*	0/192 (0)	<1	NS
ORD9922-4B	*pif1Δ sgs1Δ*	21/363 (5.8)	19	<0.01
ORT4885	*mph1Δ*	0/336 (0)	<1	NS
ORT4840	*srs2Δ*	2/276 (0.7)	2.3	NS
ORD6786-4A	*rif1Δ*	0/368 (0)	<1	NS
ORD7565-2C	*pif1Δ rad52Δ*	2/384 (0.5)	2	NS
ORD7574-9B	*pif1Δ rad51Δ*	1/384 (0.3)	1	NS
ORD7574-11C	*rad51Δ*	0/272 (0)	<1	NS

*: Fisher-test.

NS: non significant.

**Figure 2:**
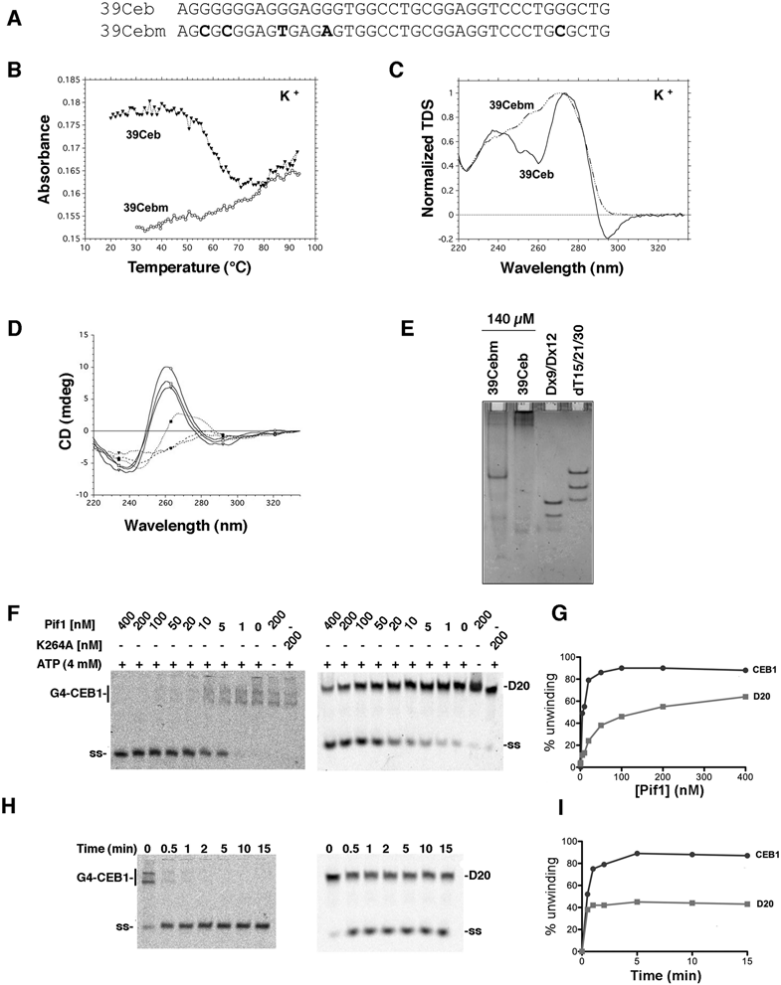
Evidence for G4 structures formation by CEB1 minisatellite sequences. (A) Oligonucleotide sequences. The 39Ceb oligonucleotide mimicks one full CEB1 repeat. 39Cebm is a control sequence with five base substitutions (shown in bold) (B) Melting profiles. Absorbance at 295 nm *vs* temperature plots for 39Ceb (triangles) and 39Cebm (circles) each at 3 µM strand concentration. Melting experiments are performed in 10 mM lithium pH 7.2 cacodylate buffer supplemented with 0.1 M KCl. (C) Thermal difference spectra. Thermal difference spectra result from the difference between the absorbance recorded at 79±2°C and at 40±2°C in a 10 mM lithium pH 7.2 cacodylate buffer supplemented with 0.1 M KCl. Thermal difference spectra are normalized (TDS_norm_ = TDS/max(TDS)) over the 220–335 nm wavelength range. Full line: 39Ceb; dotted line: 39Cebm. (D) Circular dichroism spectra. Oligonucleotides were prepared at 140 µM strand concentration and annealed in 1 M NaCl as in helicase experiments, then immediately diluted to 3 µM in a 10 mM lithium cacodylate 1 M NaCl, pH 7.2 buffer. Full line: 39Ceb; dotted line: 39Cebm. Spectra were recorded at three different temperatures: 25°C (squares), 65°C (circles) and 90°C (triangles). (E) Behavior of the 39Ceb and 39Cebm sequences on a non-denaturing gel. Oligonucleotides were prepared at 140 µM strand concentration, annealed in 1 M NaCl as in helicase experiments and loaded on a non-denaturing 15% acrylamide gel supplemented with 20 mM NaCl and run at 21°C. Migration markers are 1: double-stranded DNA (9 and 12 bp) and 2: (dT)_15_, (dT)_21_ and (dT)_30_ oligomers. (F) In vitro unwinding of 2 nM G-quadruplex DNA (G4-CEB1, left) versus 2 nM double stranded DNA oligonucleotide substrate (D20, right) in presence of decreasing amount of Pif1 (WT or helicase-dead *pif1-K264A*) for 15 minutes at 35°C in presence or absence of ATP. (G) Quantifications of the gels shown in F. (H) Kinetics of unwinding of G-quadruplex DNA (left) versus double stranded DNA oligonucleotide D20 (right) in presence of 100 nM Pif1. (I) Quantifications of the gels shown in H.

To determine if the helicase activity of Pif1 was required to stabilize the CEB1-1.8 allele, we examined the stability of CEB1-1.8 in strains carrying the *pif1-K264A* or *pif1-K264R* mutations, which inactivate Pif1 ATPase/helicase activity [28]. In both mutants, the frequency of CEB1 rearrangement was increased approximately 10-fold over the WT level (3.2%; Table 1, Figure 1B). Thus, the helicase activity of Pif1 has a role in the stabilization of the CEB1 repeats during vegetative growth. Compared to the *pif1Δ* mutant, the frequency of size variants was approximately two-fold lower in both of the helicase-inactive mutants. This suggests that while ATPase/helicase activity is totally inactive in helicase-dead *pif1-K264A* mutant (see below, Figure 2F), the *pif1-K264A* polypeptide which retains wild type level of DNA binding [24], may act within a complex of proteins sufficient to partially protect CEB1 repeats from damage or recombinational repair.

### CEB1-1.8 Rearrangements in *pif1*Δ Cells Are Often Complex and Depend on the Rad51- and Rad52-Dependent Homologous Recombination Pathway

To characterize the internal structures of CEB1-1.8 rearrangements obtained in the *pif1Δ* cells, we sequenced nine CEB1 contractions and compared them to the parental motif. As shown in Figure 1C and Figure S1, the sequenced contractions from *pif1Δ* cells were all different from each other. Three were simple deletions, one was a double deletion and five were complex events.

To determine whether or not the destabilization of CEB1-1.8 in *pif1Δ* cells was dependent on homologous recombination, we tested the stability of CEB1-1.8 in *pif1Δ rad52Δ* and *pif1Δ rad51Δ* double-mutants. In both strains, rearrangement of CEB1-1.8 occurred at close to WT levels, strongly reduced compared to *pif1Δ* cells (Figure 1D and Table 1). We conclude that the molecular events leading to CEB1 rearrangement are repaired by homologous recombination, similar to what is seen in the absence of Rad27 [25].

### CEB1 Destabilization Is Not a Secondary Effect of Telomere or Mitochondrial Defect in *pif1Δ* Cells

To determine if the effects of *pif1Δ* on CEB1 stability are a secondary consequence of the increased telomere length or mitochondrial DNA depletion that are characteristic of *pif1Δ* cells, we examined CEB1-1.8 stability in mutants that affect either telomere length or maintenance of mitochondrial DNA. The deletion of the *RIF1* gene results in telomere lengthening [29], a phenotype likely due to the enhanced access of telomerase to the telomere [30]. *RIF1* inactivation did not destabilize CEB1-1.8 (Table 1), indicating that long telomeres are not sufficient to destabilize CEB1-1.8 repeats.

Pif1 is present as two isoforms, one targeted to the nucleus and one to mitochondria. The *pif1-m1* mutation prevents the synthesis of the mitochondrial isoform, resulting in mitochondrial deficiency but leaving nuclear Pif1 functions intact. In *pif1-m2* cells, only the mitochondrial form is detected by western analysis [28], and this strain has normal mitochondrial function and long telomeres. However, telomere lengthening and de novo telomere addition are not as elevated in *pif1-m2* cells as in a *pif1Δ* strain suggesting that some nuclear function is retained in the *pif1-m2* allele [23]. As expected, CEB1-1.8 was not destabilized (1/192) in *pif1-m1* cells (Table 1). Surprisingly, CEB1 was also stable in *pif1-m2* cells (1/384) (Table 1), a result that can be explained if *pif1-m2* cells retain sufficient nuclear Pif1 to carry out its role in maintaining CEB1 stability. To test if a low level of the Pif1-m2 polypeptide could be active in the nucleus, we examined complementation of the *pif1-m2* telomere phenotype by over expressing the *pif1-m2* protein from its own promoter on a multi-copy 2 µ plasmid in *pif1Δ* cells. Telomeres were shorter in the strain over-expressing the *pif1-m2* construct than in the control *pif1Δ* cells (data not shown). These results support our interpretation that in *pif1-m2* cells, there is sufficient nuclear Pif1 protein to stabilize CEB1, although it is insufficient to sustain normal length telomeres. A similar observation was recently reported in the fission yeast *S. pombe*. As in budding yeast, the Pif1 homolog Pfh1p is present as a mitochondrial and a nuclear isoforms. However, expression of the mitochondrial-only isoform is able to complement pfh1p nuclear defects, even though the protein is not detectable in the nucleus at the protein level by western blot [31].

### CEB1-1.8 Is Not Destabilized by Mutations in Other Helicases

We investigated if the inactivation of other helicases would also affect CEB1-1.8 stability. We previously showed that in a *dna2-1* strain, CEB1-1.8 was modestly destabilized (1.8% instability) [26]. The viability of the *DNA2* deletion in combination with the deletion of *PIF1* [27] allowed us to examine the behavior of CEB1 in the complete absence of *DNA2*. As indicated in Table 1, in the *pif1Δ dna2Δ* CEB1-1.8 strain, the frequency of CEB1 size variation was estimated at 4.7%, a value significantly higher than in wild-type cells (p<0.01, Fisher's Exact Test), but not different than in the *pif1Δ* single mutant (p = 0.48, Fisher's Exact Test). This result indicates that the complete absence of Dna2 neither suppresses nor enhances the effects of Pif1 inactivation.

Next, we examined the inactivation of Rrm3, a 5′-3′ DNA helicase that is closely related to Pif1 [32]. As shown in Table 1, deletion of the *RRM3* gene did not destabilize CEB1-1.8. Moreover, the frequency of rearrangement of CEB1 was not statistically different in the *pif1Δ rrm3Δ* (3.9%) and *pif1Δ* (6.0%) cells (p = 0.2, Fisher's Exact Test).

We tested three additional helicases with well characterized roles in genome stability for the effects on CEB1-1.8 stability. We examined the RecQ homolog Sgs1 helicase involved in multiple aspects of DNA recombination and repair [33]–[38], Srs2, a 3′ to 5′ helicase that disassembles abortive recombination intermediates [39], and the Mph1 helicase that plays a role in DNA repair [40]. Inactivation of these helicases did not destabilize the CEB1-1.8 array, and the inactivation of both Pif1 and Sgs1 helicases (*pif1Δ sgs1Δ* strain), induced the same CEB1 instability as the *pif1Δ* strain (Table 1). We conclude that the role of Pif1 in stabilizing CEB1-1.8 is specific for Pif1, rather than a general function of DNA helicases involved in DNA repair or recombination.

### All Tandem Repeated Sequences Are Destabilized in *rad27Δ cells* But Only CEB1 Is Destabilized in the Absence of Pif1

**Figure 3:**
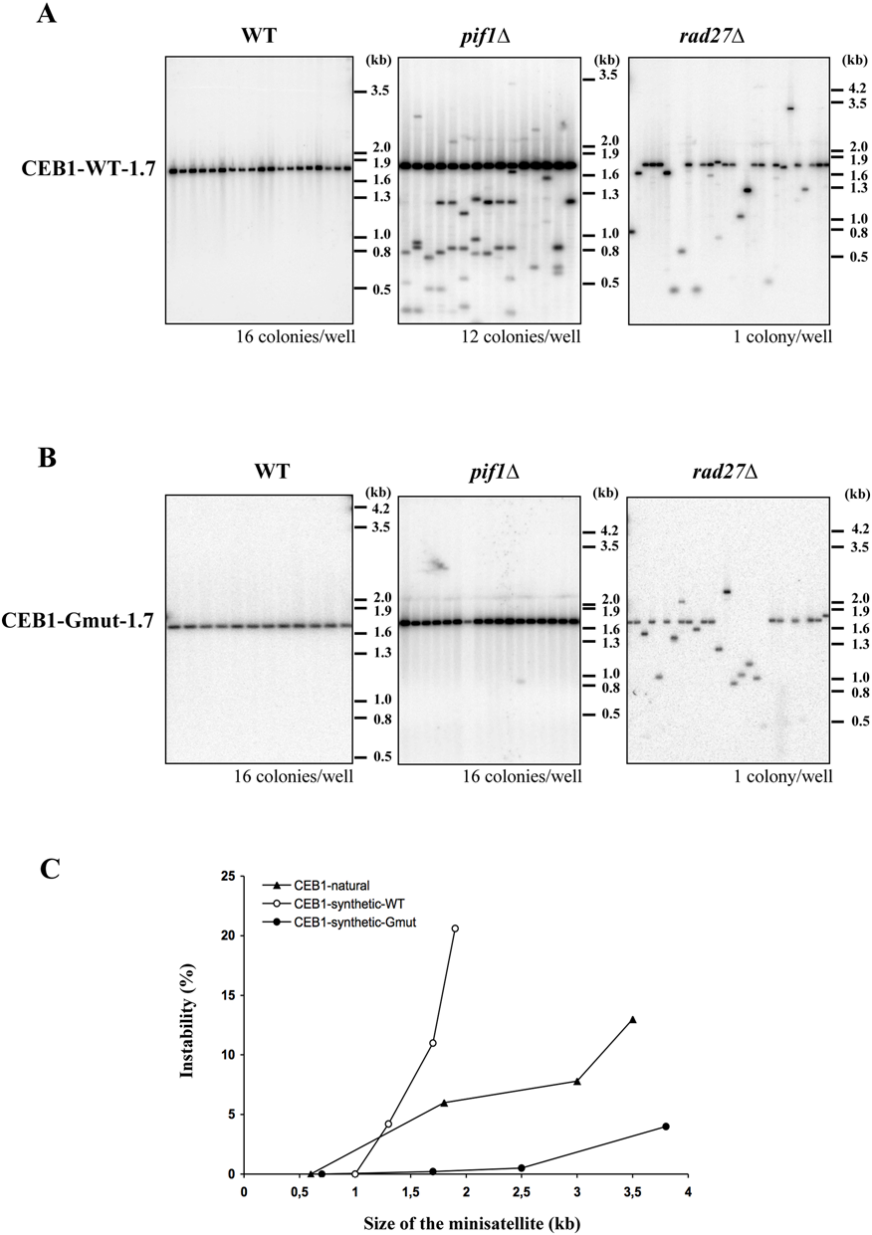
CEB1 instability in *pif1Δ* cells depends on its potential to form G4 DNA. Comparison of synthetic-CEB1-WT-1.7 (A) and synthetic-CEB1-Gmut-1.7 (B) instability in WT, *pif1Δ* and *rad27Δ* haploid strains by Southern blot analysis. In order to increase the number of independent colonies analyzed in *pif1Δ* and WT strains, colonies are pooled for DNA extraction and the number of colonies analyzed per well is indicated under each gel. DNA is digested by *Apa*I/*Spe*I and hybridized with a CEB1-WT or CEB1-Gmut probe. When several rearranged minisatellites migrate at the same size they are considered as clonal and are counted only one time. The frequency of instability for each synthetic minisatellite is reported in Table 3. (C) Frequency of natural and synthetic CEB1 minisatellites according to the size of the alleles in *pif1Δ* cells.

We examined CEB1 alleles of various sizes, a shorter allele CEB1-0.6 (14 repeats) and two longer alleles, CEB1-3.0 (65 repeats) and CEB1-3.5 (75 repeats). The two longer alleles were destabilized in *pif1Δ* cells, with instability increasing with the size of the array (Table 2, Figure 3C). For comparison, we performed similar studies in the *rad27Δ* cells. In all cases CEB1 rearrangements occurred at a lower frequency in the *pif1Δ* cells than in *rad27Δ* cells [25]. In the case of CEB1-1.8, for which the largest sample of cells was examined, its instability was approximately 5-fold higher in *rad27Δ* than in *pif1Δ* cells.

**Table 2:** Instability of various tandem repeated sequences in *pif1Δ* and *rad27Δ* cells.

Name of the tandemly repeated sequence	Size of the motif (bp)	Number of repeats	Strain	Genotype	Number of rearrangements/total (%)
CEB1-0.6	39–42	14	ORD7557-10B	*pif1Δ*	0/276 (0)
CEB1-1.8	39–42	42	ORT4841	*pif1Δ*	40/672 (6.0)
CEB1-3.0	39–42	65	ORD7598-12C	*pif1Δ*	15/192 (7.8)
CEB1-3.5	39–42	75	ORT4841-4E1	*pif1Δ*	25/192 (13.0)
*DAN4*	18	30	ORD7568	*pif1Δ*/*pif1Δ* *	0/192 (0)
			ORD6708	*rad27Δ*/*rad27Δ* *	3/52 (5.7)
*FLO1*	135	17	ORT4843	*pif1Δ*	0/192 (0)
			ORD6713-8D	*rad27Δ*	8/304 (2.7)
*HKR1*	42	21	ORT4841	*pif1Δ*	0/384 (0)
			ORD6713-8D	*rad27Δ*	11/158 (7)
*NUM1*	192	10	ORT4841	*pif1Δ*	0/384 (0)
			ORD6713-8D	*rad27Δ*	5/304 (1.6)
*hRAS1*	28	75	AND1228-2A	*pif1Δ*	0/384 (0)
			AND1228-8C	*rad27Δ*	28/55 (51)

Name of the plasmid with tandemly repeated sequence (motif)	Size of the motif (bp)	Number of repeats	Strain	Genotype	Fold increase vs. WT (frequency 10^−5^)
pMD28 (G)	1	18	ORT5604	WT	1 (0.2)
			ORT5600	*pif1Δ*	2
			ORT6009	*rad27Δ*	86400
pBK1 (GAGT)	4	16	ORT5606	WT	1 (0.4)
			ORT4896	*pif1Δ*	1.5
			ORT6014	*rad27Δ*	31200
pBK3 (CAACG)	5	15	ORT5614	WT	1 (1.8)
			ORT4894	*pif1Δ*	1
			ORT6013	*rad27Δ*	7730
pBK10 (CAATCGGT)	8	10	ORT5602	WT	1 (0.7)
			ORT4892	*pif1Δ*	1
			ORT6011	*rad27Δ*	17250
pEAS20 (CAACGCAATGCGTTGGATCT)	20	3	ORT5608	WT	1 (0.8)
			ORT4898	*pif1Δ*	3
			ORT6016	*rad27Δ*	17800

*: The repeated sequence is homozygous.

Next, we examined the instability of four natural yeast minisatellites that are normally found in the coding regions of the *DAN4*, *FLO1*, *HKR1* and *NUM1* genes [41]. This set represents a large variety of motifs in term of size (18 to 192 bp) and repeat units (10–30). All of these motifs were altered in *rad27Δ* but not in *pif1Δ* cells (Table 2). Likewise, the GC-rich hRAS1 human minisatellite [42] was not altered when propagated in *pif1Δ* cells (0/384 colonies).

Finally, using the plasmid assay developed by Kokoska *et al.* (1998), we compared the behavior of four microsatellite sequences composed of 1, 4, 5 and 8 nucleotide motifs and a triplication of a 20 nucleotides motif in wild-type, *pif1Δ* and *rad27Δ* haploid cells (see Table 2 for sequence of motifs). As previously reported [43], the rearrangement frequencies in the wild-type strain were on the order of 10^−5^–10^−6^ and were stimulated more than 10,000 fold in *rad27Δ* cells (Table 2). However, no significant increase in instability was detected in *pif1Δ* cells. Thus, in contrast to the strong and ubiquitous effects of Rad27 on minisatellite and microsatellite stability [41],[43],[44], the absence of Pif1 destabilized only the CEB1 arrays.

### The CEB1 Repeat Forms G4 Structures In Vitro

DNA oligonucleotides containing at least four successive runs of three or more guanines have been shown to fold into intramolecular G4 DNA in presence of physiological concentrations of monovalent cations [45]. Examination of the CEB1 repeat sequence revealed the presence of 3 to 5 triplets of guanines localized on the same strand in each repeat of the CEB1-1.8 allele (Figure 2A and Figure S1). It suggests that this minisatellite may form G4 structures, even if its primary sequence does not fit perfectly the d(G_3+_N_1–7_)_4_ consensus used for most bioinformatic analyses. To test this hypothesis, we examined in vitro the formation of secondary structures using a single-stranded oligonucleotide that mimicked a complete CEB1 repeat (39Ceb) or a control sequence in which five of the guanines had been mutated (39Cebm) (Figure 2A). Four complementary assays were performed to detect the formation of G4 structures:

First, 39Ceb and 39Cebm oligos were incubated in presence of 100 mM NaCl or KCl in conditions that favor G4 DNA formation. We measured the absorbance at 295 nm of 39Ceb and 39Cebm oligos at increasing temperatures. Indeed, an inverted transition corresponding to a conformational change associated with the temperature increase was observed with the 39Ceb oligo at a melting temperature (Tm) of ≈48°C in NaCl and 55°C in KCl, while no clear transition was seen with the 39Cebm sequence (Figure 2B and Table S2). Truncated versions of this motif were also analyzed (Table S2). Second, thermal differential spectra (TDS), which measure the difference between UV absorbance spectra of the oligonucleotide measured at a temperature above Tm (unfolded state) and below Tm (folded state), provides a clear signature for each type of nucleic acid structures including G4 DNA [46]. We measured the TDS in K^+^ buffer for 39Ceb and 39Cebm. As shown in Figure 2C, 39Ceb exhibits the typical pattern of a G4 structure with two positive maxima at 240 and 275 nm and a negative minimum around 295 nm [46]–[48] while 39Cebm exhibited a different signature, which does not correspond to quadruplexes.

Third, we measured the circular dichroism (CD) spectra of the two oligonucleotides under experimental conditions that mimick the helicase assays (see below; briefly oligonucleotides were incubated at 140 µM strand concentration for 48 hours in 1 M NaCl). A positive maxima around 260 nm and a negative minimum around 240 nm was observed in the CD spectra of 39Ceb, an observation in agreement with the formation of parallel G4 structures (Figure 2D) [49],[50]. In contrast 39Cebm did not exhibit a CD spectra characteristic of any G4 structure found so far. Furthermore, when prepared under these conditions, the quadruplexes were extremely stable, as shown by temperature-independent CD profiles between 25°C and 90°C. This demonstrates that these structures are extremely heat resistant (no melting transition was observed by absorbance at 295 nm when the sample was prepared with this protocol; data not shown).

Finally, 39Ceb and 39Cebm oligonucleotides were analyzed by polyacrylamide gel electrophoresis under native conditions where G4 structures are expected to show different mobility compared to unstructured oligonucleotides. No migration anomaly was found for 39Ceb when incubated in 100 mM LiCl, which does not stabilize G4 secondary structures [51] (data not shown). When 39Ceb is incubated in a sodium buffer at high strand concentration (Figure 2E; conditions identical as for helicase experiments, see below), bands of very low mobility were clearly visible. Intermolecular G4 structure formation was revealed by slow migrating bands as compared to the migration pattern of 39Cebm mutated control (Figure 2E). These higher order species likely correspond to bimolecular, tetramolecular (or higher) G4 structures. These experiments were repeated at lower strand concentration (50 nM or 4 µM), both in sodium and potassium. As expected for multimers (dimers, tetramers or species of even higher stoichiometry), concentration-dependent profiles were obtained (Figure S2).

In conclusion, in all assays, the oligonucleotides containing the G-strand of the CEB1 motif exhibited the hallmarks of G4 structure formation in vitro whereas the 39Cebm control sequence did not. Depending on buffer conditions, strand concentration and incubation protocol, a variety of different quadruplex structures could be obtained with this sequence, arguing for the possible formation of multiple quadruplexes in vivo.

### Pif1 Protein Unwinds G4 CEB1 DNA In Vitro

If CEB1 also forms G4 DNA in vivo, Pif1 might inhibit CEB1 rearrangements by unwinding these structures. The prediction of this model is that Pif1 should be able to unwind these structures. To test this prediction oligonucleotides containing one CEB1 repeat were incubated in vitro using conditions that favor the formation of intermolecular G4 structures (see Materials and Methods). The G4-DNA substrate was first incubated in the presence of decreasing amount of purified recombinant Pif1. Upon 15 minutes incubation at 35°C, 5 nM Pif1 was enough to unwind 50% of the 20 fmol (2 nM) G4-DNA, while at least 20 times more Pif1 was necessary to unwind 20 fmol (2 nM) of a double-stranded oligonucleotide substrate (Figure 2F, G). The unwinding of both substrates required Pif1 helicase activity as no unwinding is observed in absence of ATP, or when the substrate is incubated in presence of saturating amount of the *pif1-K264A* helicase-dead mutant (Figure 2F). The rate of G4-DNA unwinding was also faster than unwinding of the double-stranded DNA substrate (Figure 2H, I). Indeed, 100 nM Pif1 was able to unwind 20 fmol (2 nM) of G4-DNA substrate in less than 5 minutes, while the enzyme was only able to unwind about 40% of the double-stranded substrate over the entire time course. These results demonstrate that Pif1 is more efficient at unwinding G4-DNA structures than regular double-stranded DNA.

### Synthetic CEB1 Alleles Without G4 Prone Sequence Are Stable in *pif1Δ* Cells

The in vitro experiments demonstrating the propensity of the CEB1 repeat to form G4 structures and the ability of Pif1 to unwind these structures led us to consider that Pif1 might unwind G4 structures in CEB1 in vivo. If this model is correct, mutations in CEB1 that eliminate its ability to form G4 structures might render it insensitive to Pif1. For these experiments, we developed a method combining both in vitro and in vivo steps to construct long (>1 kb) synthetic CEB1 alleles (see Text S1). We generated two categories of synthetic CEB1 arrays based on two different repeat units. The first category, named synthetic-CEB1-WT, was based on the repetition of the most common motif of the natural polymorphic CEB1-1.8 allele (Figure S3, A, D). The second category, named CEB1-Gmut, was made from oligonucleotides in which 5 dispersed G bases were changed to either C, A or T in order to disrupt the original 5 G-triplets on the G-rich strand (Figure S3, A, E). In vitro analysis of the secondary structures of CEB1-Gmut oligonucleotides demonstrated that, as expected, they were unable to form G4 structures (39Cebm, Figure 2 and Table S2).

The rearrangement frequency of the synthetic-CEB1-WT arrays (1.0, 1.3, 1.7, 1.9 and 2.3 kb long) and of the synthetic-CEB1-Gmut arrays (0.7, 1.7, 2.5 and 3.8 kb long) in WT, *pif1Δ* and *rad27Δ* cells is reported in Table 3 and summarized in Figure 3. As observed for the natural CEB1 alleles, the rearrangement frequency of the synthetic-CEB1-WT arrays was low in WT cells and increased in a size dependent manner in both *pif1Δ* and *rad27Δ* cells. In all cases, the frequency of instability for similarly sized alleles was higher in the synthetic-CEB1-WT arrays than in the natural CEB1 alleles. We attribute this difference to the greatly reduced polymorphism of the synthetic allele. However, the most striking result was that mutations in G4 prone motifs strongly decreased the frequency of their rearrangement in *pif1Δ* cells. We observed only one rearrangement of the CEB1-Gmut-1.7 allele among the 383 colonies analyzed (0.2%) while the synthetic-CEB1-WT-1.7 allele was rearranged in 38/343 *pif1Δ* colonies (11%) (Figure 3 and Table 3). Similarly, the large synthetic-CEB1-Gmut-3.8 array, which contains approximately 97 repeats, yielded only a few rearrangements in the *pif1Δ* and WT strains (4% and 2%, respectively; this difference was not statistically different, p = 0.18, Fisher's Exact Test). In contrast, CEB1-Gmut arrays rearranged in *rad27Δ* cells and the frequency of rearrangement increased in a size dependent-manner (Table 3). Thus, the synthetic and natural CEB1 alleles behaved similarly while the artificial CEB1 arrays containing mutation of G4-prone sequences were stabilized in *pif1Δ* but not in *rad27Δ* cells. These results strongly support our proposal that formation of G4 structures within the CEB1 array is responsible for their instability in vivo and that this secondary structure is processed by the Pif1 helicase.

**Table 3:** Instability of synthetic minisatellites in WT, *pif1Δ* and *rad27Δ* cells.

Minisatellite	Number of repeats	Strain	Genotype	Number of rearrangements/total (%)
CEB1-WT-2.3	58	AND1207-9B	WT	6/384 (1.5)
CEB1-WT-1.7	44	AND1212-10D	WT	2/708 (0.3)
CEB1-WT-1.0	26	AND1213-1D	WT	0/192 (0)
CEB1-WT-1.9	48	AND1202-13D-P14C3	*pif1Δ*	32/154 (20.6)
CEB1-WT-1.7	44	AND1202-11A	*pif1Δ*	38/343 (11)
CEB1-WT-1.3	33	AND1202-11A-L8C12	*pif1Δ*	8/189 (4.2)
CEB1-WT-1.0	26	ORT6108-4	*pif1Δ*	0/192 (0)
CEB1-WT-1.7	44	AND1218-1A	*rad27Δ*	15/50 (30)
CEB1-WT-1.0	26	ORT6110-1	*rad27Δ*	32/576 (5.5)
CEB1-Gmut-3.8	97	AND1206-5D	WT	8/384 (2)
CEB1-Gmut-1.7	42	AND1227-5C	WT	0/192 (0)
CEB1-Gmut-3.8	97	AND1206-4C	*pif1Δ*	8/192 (4)
CEB1-Gmut-2.5	64	AND1206-4C-1B6-1E1	*pif1Δ*	1/192 (0.5)
CEB1-Gmut-1.7	42	AND1206-4C-D11P2	*pif1Δ*	1/383 (0.2)
CEB1-Gmut-0.7	19	ORT6107-1	*pif1Δ*	0/192 (0)
CEB1-Gmut-3.8	97	AND1206-4B	*rad27Δ*	35/51 (68.6)
CEB1-Gmut-1.7	42	AND1226-18B/-17C	*rad27Δ*	52/98 (53)
CEB1-Gmut-0.7	19	ORT6109	*rad27Δ*	23/552 (4.1)

## Discussion

In the present study, we provide new insights into the biochemical and biological functions of the evolutionary conserved Pif1 helicase. Our main findings are: (i) inactivation of Pif1 increased the frequency of rearrangement of the G-rich CEB1-1.8 tandem array, (ii) this increased rearrangement was specific for Pif1 as mutation of other helicases did not affect the stability of CEB1 and other repeats were stable in *pif1Δ* cells, (iii) the G-rich strand of the CEB1 repeat unit formed G-quadruplex structures in vitro, (iv) Pif1 readily unwound the CEB1 G4 structures in vitro and, (vi) mutation of the G4–forming motifs stabilized CEB1 in *pif1Δ* cells. Destabilization of CEB1 in *pif1Δ* cells was not an indirect consequence of other *pif1Δ* phenotypes such as respiratory deficiency or long telomeres. Thus, the experiments reported here uncover a new activity for the Pif1 helicase, the ability to process G4 secondary structures, and suggest that this activity contributes to genome stability by preventing the rearrangement of G4 forming repeats in vivo.

### Mechanism of CEB1 Repeats Instability

In previous studies, we reported that human CEB1 repeats inserted into the yeast genome are highly unstable in absence of the Rad27 endonuclease and slightly unstable in a *dna2-1_ts_* mutant [25],[26]. Since Rad27 and Dna2 are involved in the processing of flap structures during Okazaki fragment maturation [52], we concluded that CEB1 instability was likely due to the accumulation of unresolved flap structures during replication. We proposed that these intermediates would form recombinogenic structures that are repaired by homology-dependent strand displacement and annealing (SDSA) [53].

Here we show that inactivation of Pif1 also resulted in CEB1 instability. As in *rad27Δ* cells, the CEB1 rearrangements in *pif1Δ* cells had a high frequency of complex events (Figure 1C; [25]). In addition, in both mutants, CEB1 rearrangements depended on Rad52/Rad51-dependent homologous recombination (Table 1). These similarities suggest that the repair of the lesion leading to CEB1 rearrangement in the absence of either Pif1 or Rad27 occurs by SDSA, although the recombinogenic lesion may be different (for example a single-strand gap or a double-strand break). In *pif1Δ* and *rad27Δ* cells, the frequency of rearrangements increased with the size of the allele (Figure 3C; [25]). In *rad27Δ* cells, this increased instability may reflect the increased probability that longer arrays are more likely to contain more than one improperly processed flap. Similarly, in *pif1Δ* cells, long CEB1 minisatellites could form G4 structures with a higher probability, especially if quadruplexes involve G-tracts from adjacent repeats. Alternatively, lesions in small alleles could be rare or more often resected into the non-repeated flanking sequences, leading to the preferential restoration of the parental sequence by homologous recombination in G2 cells using the intact sister chromatid as a template [53].

### CEB1 Repeats Are Unstable in *pif1Δ* Cells Only if They Are Able to Form G4 Structures

**Figure 4:**
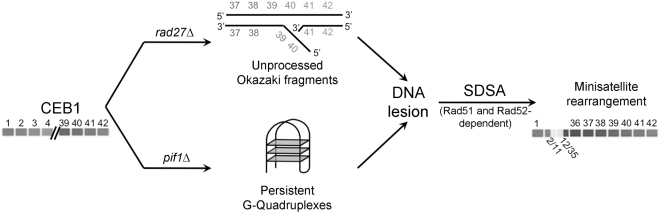
Proposed model for CEB1 rearrangements in *rad27Δ* and *pif1Δ* mutants. In the absence of Rad27, accumulation of unresolved flap structures inside CEB1 during replication generates recombinogenic structures that are repaired by homology-dependent strand displacement and annealing (SDSA). While in the absence of Pif1, the persistence of unprocessed G-quadruplex secondary structures in CEB1, during replication, transcription or other processes, initiates DNA lesions that are also repaired by SDSA, leading to minisatellite rearrangements.

Whereas all micro- and minisatellites sequences tested are unstable in *rad27Δ* cells ([43],[44] this study), only CEB1 was unstable in *pif1Δ* cells (Table 2). The CEB1 sequence is G/C rich (72%) with a high strand bias (23 G and 7 C per repeat of 39 bases). However, the instability of CEB1 in *pif1Δ* cells can not be attributed solely to its G/C rich sequence as the human hRAS1 minisatellite, which is also G rich (68%) with a strong bias (14 G and 5 C per repeat of 28 bases), was stable in the absence of Pif1. Each CEB1 repeat contains putative G4 signature motifs. Our biophysical analyses of CEB1 and hRAS oligonucleotides showed that the CEB1 motif readily formed G4 structures in vitro while hRAS1 did not (Figure 2 and Table S2). Moreover, synthetic CEB1 minisatellites in which the runs of guanine were mutated to disrupt their ability to form G4 structures were no longer unstable in *pif1Δ* cells. We propose that the recombinogenic lesions formed in the absence of Pif1 are unresolved intra- or inter-motifs G4 structures. Thus, while CEB1 alleles are unstable in both *pif1Δ* and *rad27Δ* cells, the events that initiate instability, unprocessed Okazaki fragments (in *rad27Δ* cells) or persistent G4 structures (in *pif1Δ* cells) are different (Figure 4). As a result, all tandem arrays are unstable in the absence of Rad27, including the synthetic G4-mutated CEB1 alleles, while only CEB1 was unstable in *pif1Δ* cells.

### In Vivo Roles of Pif1

What do our results suggest about the role(s) of Pif1 in the cell? Owing to the alternative use of a translation start site, *PIF1* generates two isoforms, one with mitochondrial and one with nuclear functions. Several observations indicate that Pif1 is involved in the maintenance of mitochondrial DNA. Specifically, Pif1 increases the frequency of recombination between ρ^+^ and certain ρ^−^ tandemly repeated mitochondrial genomes [21]. The loss of Pif1 is thought to trigger mtDNA breakage in specific regions, leading the authors to propose that Pif1 recognizes a specific but uncharacterized DNA topology [22],[54]. Although the ∼75 kb *S. cerevisiae* mitochondrial genome is AT-rich, it contains numerous G-rich stretches. We speculate that in the absence of mitochondrial Pif1, breaks occur due to defective processing of G4 structures and these breaks are repaired by recombination. Alternatively, G4 DNA can create a structural target for factors involved in DNA recombination.

In the nucleus, Pif1 affects telomere length through direct inhibition of telomerase [23],[28] the specialized reverse transcriptase that lengthens telomeres in most eukaryotes. In vivo and in vitro data suggest that telomerase inhibition is achieved by direct displacement of telomerase from a DNA end [24]. Since Pif1 exhibits a marked preference for RNA-DNA hybrid unwinding in vitro [55], Pif1 is proposed to inhibit telomerase by unwinding the RNA-DNA hybrid formed between the telomerase RNA, TLC1, and the telomeric DNA end. Pif1-mediated removal of telomerase from DNA ends can explain the effects of *pif1* mutations on both telomere length and de novo telomere addition [23],[56] as well as its inhibition of gross chromosomal rearrangements [57]. Human Pif1 (hPIF) may have similar functions as ectopic expression of hPIF causes telomere shortening and decreased telomerase processivity in vitro [58]. In addition, hPIF co-immunoprecipitates with telomerase subunits and telomerase activity [59]. Importantly for the present study, most telomeric DNA sequences, including yeast and human telomeric DNA, can form G4 structures in vitro. Moreover, G4 structures have been detected at ciliate telomeres in vivo [6]. In budding yeast, no evidence of the presence of G4 structures in the telomeric single stranded region has yet been reported, but proteins that bind or process G4 DNA in vitro are nevertheless present at yeast telomeres. In particular, in vitro studies have shown that the telomere binding protein Rap1 binds double-stranded telomeric DNA and promote the formation of G-quadruplex structures [60]. It is not known if this reaction occurs in vivo, but it is tempting to speculate that the formation of G4 DNA is necessary to promote the assembly of functional telomere. Alternatively or in addition to its ability to inhibit telomerase directly, Pif1 could counteract the formation of G4 structures in telomeric DNA, thus antagonizing the formation of proper telomere architecture. Consistent with this hypothesis, it has been shown that Pif1 overexpression compromises the viability of yeast strains with compromised telomere end protection [61].

Several studies suggest that Pif1 also has non-telomeric roles in replication and repair of nuclear DNA. First, in the rDNA, Pif1 helps maintain the replication fork barrier during replication [32]. Second, Pif1 is recruited to Rad52 DNA repair foci after gamma irradiation [62]. Third, lack of Pif1 suppresses the lethality of a Dna2 deletion, a helicase/endonuclease involved in the processing of Okazaki fragments by removing long 5′ flaps. Although the role of Pif1 in Okazaki fragment maturation is unclear, it is proposed to act by extending the flaps created by the lagging strand replicative polymerase at the junction of two consecutive Okazaki fragments [27]. Like Pif1, Dna2 is involved in telomere maintenance [63] and is able to process G4 DNA in vitro [64]. Thus, the two enzymes may act in concert to remove toxic intermediates, including G4-DNA, which could arise during lagging strand replication and, if not appropriately processed, promote formation of recombinogenic DNA lesions, such as double strand breaks.

Finally, considering that in addition to G4-unwinding, Pif1 more efficiently unwinds RNA/DNA hybrids than DNA/DNA substrates [55], it is also to be envisaged that Pif1 plays a more general role in yeast cells when potential G4 structure can form, for example, during transcription.

### Multiplicity and Specificity of G4-Processing Helicases

Budding yeast as well as all the other organisms encodes a large number of helicases. Current estimate in *S. cerevisiae* is approximately 120. This multiplicity raises the question of their specific substrate(s) and function(s), an issue which remains often unresolved and controversial. In *S. cerevisiae*, the RecQ homolog Sgs1 helicase was proposed to resolve G4 DNA, a conclusion primarily based on its ability, and more generally of members of the RecQ family, to resolve G4 DNA structures in vitro [16]. Compelling evidence for the involvement of Sgs1 in G4 DNA metabolism in vivo finally came from the survey of global gene expression analysis in absence of Sgs1 [11]. The authors found that the set of genes which expression level is affected in *sgs1* mutant is biased towards genes that contain potential G4 forming sequences in their ORFs. To our surprise, the deletion of *SGS1* had no effect on CEB1 stability (Table 1). The lack of in vivo redundancy between Sgs1 and Pif1 in this novel assay is interesting and allows several hypotheses. First, it is possible that Sgs1 and Pif1 do not recognize the same set of G4 structures. G4 forming sequences can give rise to secondary structures exhibiting very diverse sizes, topologies (parallel or anti-parallel) and arrangements (intra- or inter-molecular) [65], and these structures may be recognized or processed differently depending on helicase. Second, Sgs1 may not recognize the G4 substrates generated by CEB1 in vivo due to the polarity of the single strand region flanking the G4-DNA structure (Pif1 is a 5′-3′ helicase while Sgs1 has a 3′-5′ polarity). Third, it is likely that the numerous repeats in CEB1 that contain G4 forming sequences lead to the formation of highly stable structures in vivo that only some helicases are able to unwind. Finally, in the absence of more direct evidences for Sgs1 involvement in G4 DNA unwinding in vivo, there is also a possibility that Sgs1 plays a minor role in maintaining G4 DNA forming sequences. In multicellular organisms, the relationships between genomic instability, G-quadruplex structures and helicases functions have also been suspected. Studies in human cells deficient for the Werner, Bloom and RTEL helicases showed defects in telomere maintenance in vivo while G4 DNA is highly suspected to form at mammalian telomeres [66],[67] and a recent study reports the correlation between genomic stability and G4 DNA unwinding by the human FANCJ helicase [18]. Similarly, in *Caenorhabditis elegans*, the disruption of the RTEL homolog DOG-1 triggers deletions of polyguanine tracts matching the G4 DNA signature [20].

Finally, it should be mentioned that the inactivation of the potential Pif1 homolog in mice has no detectable phenotype, in particular regarding change in telomere length homeostasis [68]. In light of our present study, the stability of other repeated potentially G4 forming sequences in mice and mammalian cells should be examined. Also, taking advantage of the present yeast system allowing to test natural and synthetic substrates, we anticipate that further studies of *pif1Δ* cells will allow to uncover the multiple roles of this evolutionary conserved helicase, facilitate the characterization of G4 structures in vivo and finally enhance our understanding of the dynamics of G4 formation and function in vivo.

## Materials and Methods

### Yeast Strains

The relevant genotypes and sources of haploid and diploid *S. cerevisiae* strains (S288C background) used in this study are indicated in Table S1.

### Identification of Minisatellite Rearrangements

Examination of CEB1 instability during vegetative growth was done as previously described [25]. Individual colonies or colonies pools were analyzed by Southern blot depending on the rearrangement frequency (for rearrangement frequency >20%, individual colonies were privileged). Southern blots were performed using *Alu*I digestion for natural CEB1 minisatellites and *Apa*I/*Spe*I for synthetic minisatellites and the corresponding membranes were hybridized with the radiolabeled CEB1-0.6 and CEB1-synthetic probes, respectively. For the analysis of the yeast minisatellite instability (*DAN4*, *FLO1*, *HKR1* and *NUM1*), Southern blots were performed using *Alu*I digestion (which does not cut in these repeats). Membranes were hybridized with the radiolabeled purified PCR product of the corresponding minisatellite (primer sequences available under request). For the analysis of the human *hRAS1* minisatellite instability, Southern blots were performed using *Apa*I*/Spe*I digestion and *hRAS1* probe obtained from the p37Y8 plasmid (gift from D. Kirkpatrick). Detection of signals was done with a Storm PhosphorImager (Molecular Dynamics). For pools of genomic DNA from 12 or 16 colonies/wells, rearrangement is counted when the intensity of the rearranged minisatellite, quantified with ImageQuant software, corresponds to 1/12 or 1/16 of the total amount of signals measured in the lane. When several rearranged minisatellites migrate at the same size they are considered as clonal and are counted only once.

### Sequencing of CEB1 Alleles

The internal structure of rearranged alleles was determined by DNA sequencing as described previously [25].

### Analysis of G-Quadruplex Secondary Structure

Oligonucleotides were synthesized by Eurogentec (Belgium). Concentrations of all oligodeoxynucleotides were estimated using extinction coefficients provided by the manufacturer and calculated with a nearest neighbor model [69] under low salt conditions at 60°C in order to destabilize quadruplex formation. The sequences studied are shown in Table S2. Oligonucleotides chosen for non denaturing gel electrophoresis were first purified under denaturing conditions.

Melting experiments were conducted as previously described [70]. Denaturation was followed by recording the absorbance at 240 or 295 nm [47],[71]. Melting experiments were typically performed at a concentration of 4 µM per strand. Thermal difference spectra (TDS) were obtained by difference between the absorbance spectra from unfolded and folded oligonucleotides that were respectively recorded much above and below its melting temperature (T_m_).

Circular dichroism (CD) spectra were recorded on a JASCO-810 spectropolarimeter using a 1 cm path length quartz cuvette in a reaction volume of 580 µl. Oligonucleotides were either *i)* prepared as a 4 µM solution in 10 mM lithium cacodylate pH 7.2, 100 mM NaCl or KCl buffer and annealed by heating to 90°C for 2 min, followed by cooling to 20°C or *ii)* preincubated for 48 hours at higher strand concentration (140 µM) in a 10 mM lithium cacodylate pH 7.2, 1 M NaCl buffer. Scans were performed at 25°C to 90°C over a wavelength range of 220–335 nm with a scanning speed of 500 nm/min, a response time of 1 s, 1 nm pitch and 1 nm bandwidth.

Formation of G4-DNA was confirmed by non-denaturing PAGE. In this case, oligonucleotides were either directly observed by UV shadow (when incubated at high strand concentration) or 5′ labeled with T4 polynucleotide kinase. Prior to the incubation, the DNA samples were heated at 90°C for 10 min and slowly cooled (2 h) to room temperature (or 60°C for 48 hours). Oligonucleotides were first treated with 50 mM LiOH (to unfold quadruplexes) for 10 minutes followed by HCl neutralization. Samples were incubated at 10 nM or 4 µM strand concentration in Tris-HCl 10 mM pH 7.5 buffer with 100–1000 mM Li^+^ or K^+^. 10% sucrose was added just before loading. Oligothymidylate markers (dT_15_, dT_21_, or dT_30_) or double-stranded markers (Dx_9_: 5′d-GCGATACGG+5′d-CCGATACGC Dx_12_: 5′d-GCGTGACTTCGG+5′d-CCGAAGTCACGC) were also loaded on the gel.

### Analysis of G-Quadruplex Unwinding by Pif1 In Vitro

Recombinant Pif1 was purified to homogeneity by affinity chromatography as described [55]. A Cy5-labeled oligonucleotide containing a 5′ poly(dA) tail followed by a CEB1 repeat (5′-Cy5-AAAAAAAAAAAGGGGGAGGGAGGGTGGCCTGCGGAGGTCCCTGGGCTG) was synthesized by Eurogentec (Belgium). For formation of the G-quadruplex, a solution of CEB1 oligo at 140 µM in 1 M NaCl was denatured 5 min at 100°C, then incubated at 65°C for 48 hours to promote formation of G4 intermolecular structures [72]. The double-stranded DNA control was made by annealing a 5′-Cy5-labeled 20 mer oligonucleotide to a 40 mer oligonucleotide, leaving a 20 nucleotide-long 5′ single-stranded DNA overhang. Briefly, 10 µM of each oligonucleotide were mixed in a buffer containing 10 mM Tris pH 8.0 and 5 mM Mg^2+^. The mixture was denatured 5 minutes at 95°C and slowly let to cool to room temperature. The double-stranded DNA substrate was further purified from non annealed single-stranded DNA on a MiniQ anion exchange column.

Helicase assays were carried out by incubating indicated amounts of Pif1 and 2 nM nucleic acid substrate at 35°C. Standard reaction buffer was 20 mM Tris pH 7.5, 50 mM NaCl, 100 µg/ml bovine serum albumin, 2 mM DTT, 5 mM Mg^2+^ and 4 mM ATP. For kinetic studies, reactions were started by addition of ATP in presence of 100 nM Pif1 and 2 nM substrate. 10 µl aliquots were withdrawn at indicated times and the reactions stopped by addition of 2 µl deproteinizing/loading buffer (6% Ficoll, 50 mM EDTA pH 8.0, 2.5 µg/µl Proteinase K) and incubated further 15 minutes at 35°C. Reaction products were loaded on a 10% polyacrylamide non-denaturing gel and resolved by electrophoresis at 4°C and 10 V/cm in TBE 1× buffer. Gels were dried and scanned with a storm PhosphorImager (Molecular Dynamics) and quantified using ImageQuant software (GE Healthcare).

### Statistical Analysis

Fisher exact test was performed using R software [73].

## Supporting Information

Figure S1 Sequences of the G-strand of CEB1-1.8 parental allele and of nine rearrangements obtained in the *pif1*Δ haploid strain (ORT4841). Polymorphic DNA bases are highlighted. The numbers at right in parentheses indicate the corresponding repeat in the parental CEB1-1.8 allele. Two numbers separated by dash represent hybrid repeats. Junction regions, which are delimited by polymorphisms of CEB1-1.8 derived from repeats involved in the deletions/duplications, are shaded in grey. X indicates a repeat of unknown origin or which cannot be attributed to a specific repeat in the parental CEB1-1.8 allele.
(1.66 MB PDF)

Figure S2 Behavior of the 39Ceb and 39Cebm sequences on a non-denaturing gel. Two strand concentrations were tested: radiolabeled only (around 50 nM) or supplemented with 4 µM of cold oligonucleotide. Samples were treated with 50 mM LiOH to unfold quadruplexes, reannealed in 1 M NaCl buffer (top) or KCl (bottom) for 2 hours and loaded on a non-denaturing 15% acrylamide gel and run at 26°C. Migration markers are double-stranded DNA (9 and 12 bp) and (dT)_15_, (dT)_21_ and (dT)_30_ oligomers.
(0.11 MB PDF)

Figure S3 Synthesis of artificial CEB1 minisatellites by PCR. (A) Nucleotide sequence of the CEB1-WT and CEB1-Gmut motifs. Repeats of at least three consecutive guanines are highlighted in grey in the CEB1-WT motif. Point mutations interrupting the G-triplets in the CEB1-Gmut motif are underlined. (B) Schematic representation of CEB1-concatemers synthesized by PCR. Two complementary oligonucleotides for CEB1-Gmut are represented (up and low), each composed of two identical CEB1-Gmut motifs (see Text S1 for sequences). After the first cycle of denaturation and annealing, the oligonucleotides can perfectly anneal along the two motifs and no elongation is possible (left), or they can shift and only one motif is annealed and the second motif is used as DNA template for elongation (right) resulting in addition of one motif at the end of the cycle. (C) After 30 cycles, DNA is deposited in agarose gel and the smear corresponds to a population of CEB1-concatemers of various sizes. White square indicates the part of the gel that will be cut in order to extract DNA and clone it in pGEM-T Easy vector. Sequences of the synthetic minisatellites, CEB1-WT-1.0 (D) and CEB1-Gmut-1.7 (E), with 26 and 42 repeats respectively. The sequence of the parental motif (CEB1-WT or CEB1-Gmut) used for the synthesis is indicated above the sequence of the synthetic minisatellite. Mutations and small deletions introduced during the concatemer synthesis are highlighted in red and in grey, respectively.
(0.51 MB PDF)

Table S1 List of strains used in this study.
(0.10 MB PDF)

Table S2 Sequence of the oligonucleotides used and their respective melting temperatures.
(0.08 MB PDF)

Text S1 Supplementary material and methods.
(0.13 MB PDF)
