# Supplementary material for: The Yeast Pif1 Helicase Prevents Genomic Instability Caused by G-Quadruplex-Forming CEB1 Sequences In Vivo
Source: PLoS Genet. 2009 May 8;5(5):e1000475. doi: 10.1371/journal.pgen.1000475 (PMC2673046; doi:10.1371/journal.pgen.1000475)
Supplement: Figure S1 — Sequences of the G-strand of CEB1-1.8 parental allele and of nine rearrangements obtained in the pif1Δ haploid strain (ORT4841). Polymorphic DNA bases are highlighted. The numbers at right in parentheses indicate the corresponding repeat in the parental CEB1-1.8 allele. Two numbers separated by dash represent hybrid repeats. Junction regions, which are delimited by polymorphisms of CEB1-1.8 derived from repeats involved in the deletions/duplications, are shaded in grey. X indicates a repeat of unknown origin or which cannot be attributed to a specific repeat in the parental CEB1-1.8 allele. (1.66 MB PDF) [file pgen.1000475.s001.pdf]

Figure S1

CEB1-1.8

```
GGGGGGAGGGAGGGAGGGAGGGA
    GGGAGGGTGGGAGGGTGGCCTGCCGAGGTCCCTGGGCT-A        (42)
        AGAGGGGAGAGTGGCCTGCCGAGGTCCCTGGGCTGA        (41)
     GGGGGGAGGGAGAGTGGCCTGCGGAGGTCCCTGGGCTGA        (40)
     GGGGGGAGGGAGGGTGGCCTGCGGAGGTCCCTGGGCTGA        (39)
 GGGGGGAGGGAGGGAGGGTGGCCTGCGGAGGTCCCTGGGCTGA        (38)
    GGGGGGAGGGGAGAGTGGCCTGCAGAGGTCCCTGGGCTGA        (37)
     GGGGGGAGGGAGAGTGGCCTGCAGACATCCCTGGGCTGA        (36)
 GGGAGGAGGGAGGGAGGGTGGCCTGCAGAGGTCCCTGGGCTGA        (35)
 GGGGGGAGGGAGGGAGGGTGGCCTGCGGAGGTCCCTGGGCTGA        (34)
 GGGGGGAGGGAGGGAGGGTGGCCTGCGGAGGTCCCTGGGCTGA        (33)
     GGGGGGAGGGAGGGAGGCCTGCGGAGGTCCCTGGGCTGA        (32)
     GGGGGGAGGGAGGGTGGCCTGCCGAGGTCCCTGGGCTGA        (31)
     GGGGGGAGGGAGAGTGGCCTGCAGACATCCCTGGGCTGA        (30)
 GGGAGGAGGGAGGGAGGGTGGCCTGCAGAGGTTCCTGGGTTGA        (29)
     GGGGGGAGGGAGGGTGGCCTGCAGAGGTCCCTGGGCTGA        (28)
     GGGGGGAGGGAGGGTGGCCTGCGGAGGTCCCTGGGCTGA        (27)
     GGGGGGAGGGAGGGAGGCCTGCGGAGGTCCCTGGGCTGA        (26)
    GGGGGGAGGGGAGAGTGGCCTGCCGAGGTCCCTGGGCTGA        (25)
 GGGGGGAGGGAGGGAGGGTGGCCTGCAGACATCCCTGGGCTGA        (24)
 GGGAGGAGGGAGGGAGGGTGGCCTGCAGAGGTTCCTGGGTTGA        (23)
     GGGGGGAGGGAGGGTGGCCTGCGGAGGTCCCTGGGCTGA        (22)
     GGGGGGAGGGAGGGTGGCCTGCAGAGGTCCCTGGGCTGA        (21)
 GGGGGGAGGGAGGGAGGGTGGCCTGCGGAGGTCCCTGGGCTGA        (20)
     GGGGGGAGGGAGGGTGGCCTGCAGAGGTCCCTGGGCTGA        (19)
     GGGGGGAGGGAGGGTGGCCTGCGGAGGTCCCTGGGCTGA        (18)
 GGGGGGAGGGAGGGAGGGTGGCCTGCGGAGGTCCCTGGGCTGA        (17)
 GGGGGGAGGGAGGGAAGGTGGCCTGCAGAGGTCCCTGGGCTGA        (16)
     GGGGGGAGGGAGGGTGGCCTGCAGAGGTCCCTGGGCTGA        (15)
     GGGGGGAGGGAGGGTGGCCTGCGGAGGTCCCTGGGCTGA        (14)
 GGGGGGAGGGAGGGAGGGTGGCCTGCGGAGGTCTCTGGGCTGA        (13)
 GGGGGGAGGGAGGGAAGGTGGCCTGCGGAGGTCCCTGGGCTGA        (12)
     GGGGGGAGGGAGGGTGGCCTGCGGAGGTCCCTGGGTTGA        (11)
      GGGGGAGGGAGGGTGGCCTGCAGAGGTCCCTGGGCTGA        (10)
     GGAGGGAGGGAGGGTGGCCTGCGGAGGTCCCTGGGCTGA        (9)
 GGGGGGAGGGAGGGAGGGTGGCCTGCCGAGGTCCCTGGGCTGA        (8)
     GGGGGGAGGGAGGGTGGCCTGCCGAGGTCCCTGGGCTGA        (7)
 GGGAGGAGGGAGGGAGGGTGGCCTGCGGAGGTCCCTGGGCTGA        (6)
     GGGGGGAGGGAGGGTGGCCTGCGGAGGTCCCTGGGCTGA        (5)
     GGGGGGAGGGAGGGTGGCCTGCGGAGGTCCCTGGGCTGA        (4)
     GGGGGGAGGGAGGGTGGCCTGCGGAGGTCCCTGGGCTGA        (3)
     GGGGGGAGGGAAGGTGGCCTGCGGAGGTCCCTGGGCTGA        (2)
      GGGGGAGGGAGGGTGGCCTGCAGACGTCCCT-GGCTGA        (1)
```

P8

```
GGGGGGAGGGAGGGAGGGAGGGAGGGAGGGTGGGAGGGTGGCCTGCCGAGGTCCCTGGGCTGA  (42-8)
                          GGGGGGAGGGAGGGTGGCCTGCCGAGGTCCCTGGGCTGA  (7)
                      GGGAGGAGGGAGGGAGGGTGGCCTGCGGAGGTCCCTGGGCTGA  (6)
                          GGGGGGAGGGAGGGTGGCCTGCGGAGGTCCCTGGGCTGA  (5)
                          GGGGGGAGGGAGGGTGGCCTGCGGAGGTCCCTGGGCTGA  (4)
                          GGGGGGAGGGAGGGTGGCCTGCGGAGGTCCCTGGGCTGA  (3)
                          GGGGGGAGGGAAGGTGGCCTGCGGAGGTCCCTGGGCTGA  (2)
                           GGGGGAGGGAGGGTGGCCTGCAGACGTCCCT-GGCTGA  (1)
```

P9A

```
GGGGGGAGGGAGGGAGGGAGGGAGGGAGGGTGGGAGGGTGGCCTGCCGAGGTCCCTGGGCT-A  (42)
                             AGAGGGGAGAGTGGCCTGCCGAGGTCCCTGGGCTGA  (41)
                          GGGGGGAGGGAGAGTGGCCTGCGGAGGTCCCTGGGCTGA  (40)
                          GGGGGGAGGGAGGGTGGCCTGCGGAGGTCCCTGGGCTGA  (39-40)
                          GGGGGGAGGGAGGGTGGCCTGCGGAGGTCCCTGGGCTGA  (39-7)
                      GGGAGGAGGGAGGGAGGGTGGCCTGCGGAGGTCCCTGGGCTGA  (6-4)
                          GGGGGGAGGGAGGGTGGCCTGCGGAGGTCCCTGGGCTGA  (3)
                          GGGGGGAGGGAAGGTGGCCTGCGGAGGTCCCTGGGCTGA  (2)
                           GGGGGAGGGAGGGTGGCCTGCAGACGTCCCT-GGCTGA  (1)
```

P9B

```
GGGGGGAGGGAGGGAGGGAGGGAGGGAGGGTGGGAGGGTGGCCTGCCGAGGTCCCTGGGCT-A  (42)
                             AGAGGGGAGAGTGGCCTGCCGAGGTCCCTGGGCTGA  (41-8)
                          GGGGGGAGGGAGGGTGGCCTGCCGAGGTCCCTGGGCTGA  (7)
                      GGGAGGAGGGAGGGAGGGTGGCCTGCGGAGGTCCCTGGGCTGA  (6)
                          GGGGGGAGGGAGGGTGGCCTGCGGAGGTCCCTGGGCTGA  (5)
                          GGGGGGAGGGAGGGTGGCCTGCGGAGGTCCCTGGGCTGA  (4)
                          GGGGGGAGGGAGGGTGGCCTGCGGAGGTCCCTGGGCTGA  (3)
                          GGGGGGAGGGAAGGTGGCCTGCGGAGGTCCCTGGGCTGA  (2)
                           GGGGGAGGGAGGGTGGCCTGCAGACGTCCCT-GGCTGA  (1)
```

P10

```
GGGGGGAGGGAGGGAGGGAGGGAGGGAGGGTGGGAGGGTGGCCTGCCGAGGTCCCTGGGCT-A  (42)
                             AGAGGGGAGAGTGGCCTGCCGAGGTCCCTGGGCTGA  (41)
                          GGGGGGAGGGAGAGTGGCCTGCGGAGGTCCCTGGGCTGA  (40)
                          GGGGGGAGGGAGGGTGGCCTGCGGAGGTCCCTGGGCTGA  (39)
                      GGGGGGAGGGAGGGAGGGTGGCCTGCGGAGGTCCCTGGGCTGA  (38)
                         GGGGGGAGGGGAGAGTGGCCTGCAGAGGTCCCTGGGCTGA  (37)
                          GGGGGGAGGGAGAGTGGCCTGCAGACATCCCTGGGCTGA  (36)
                      GGGAGGAGGGAGGGAGGGTGGCCTGCAGAGGTCCCTGGGCTGA  (35-12)
                          GGGGGGAGGGAGGGTGGCCTGCGGAGGTCCCTGGGTTGA  (11-2)
                           GGGGGAGGGAGGGTGGCCTGCAGACGTCCCT-GGCTGA  (1)
```

P12

```
GGGGGGAGGGAGGGAGGGAGGGAGGGAGGGTGGAGGGTGGCCTGCCGAGGTCCCTGGGCT-A  (42)
                          AGAGGGGAGAGTGGCCTGCCGAGGTCCCTGGGTTGA  (41-11)
                        GGGGGAGGGAGGGTGGCCTGCAGAGGTCCCTGGGCTGA  (10)
                       GGAGGGAGGGAGGGTGGCCTGCGGAGGTCCCTGGGCTGA  (9-4)
                       GGGGGGAGGGAGGGTGGCCTGCGGAGGTCCCTGGGCTGA  (3-8)
                       GGGGGGAGGGAGGGTGGCCTGCCGAGGTCCCTGGGCTGA  (7)
                   GGGAGGAGGGAGGGAGGGTGGCCTGCGGAGGTCCCTGGGCTGA  (6)
                       GGGGGGAGGGAGGGTGGCCTGCGGAGGTCCCTGGGCTGA  (5)
                       GGGGGGAGGGAGGGTGGCCTGCGGAGGTCCCTGGGCTGA  (4)
                       GGGGGGAGGGAGGGTGGCCTGCGGAGGTCCCTGGGCTGA  (3)
                       GGGGGGAGGGAAGGTGGCCTGCGGAGGTCCCTGGGCTGA  (2)
                        GGGGGAGGGAGGGTGGCCTGCAGACGTCCCT-GGCTGA  (1)
```

P15

```
GGGGGGAGGGAGGGAGGGAGGGAGGGAGGGTGGAGGGTGGCCTGCCGAGGTCCCTGGGCT-A  (42)
                          AGAGGGGAGAGTGGCCTGCGGAGGTCCCTGGGCTGA  (41-14)
                   GGGGGGAGGGAGGGAGGGTGGCCTGCGGAGGTCTCTGGGCTGA  (13)
                   GGGGGGAGGGAGGGAAGGTGGCCTGCGGAGGTCCCTGGGCTGA  (12)
                       GGGGGGAGGGAGGGTGGCCTGCGGAGGTCCCTGGGTTGA  (11)
                        GGGGGAGGGAGGGTGGCCTGCAGAGGTCCCTGGGCTGA  (10)
                       GGAGGGAGGGAGGGTGGCCTGCGGAGGTCCCTGGGCTGA  (9)
                   GGGGGGAGGGAGGGAGGGTGGCCTGCCGAGGTCCCTGGGCTGA  (8)
                       GGGGGGAGGGAGGGTGGCCTGCCGAGGTCCCTGGGCTGA  (7)
                   GGGAGGAGGGAGGGAGGGTGGCCTGCGGAGGTCCCTGGGCTGA  (6)
                       GGGGGGAGGGAGGGTGGCCTGCGGAGGTCCCTGGGCTGA  (5)
                       GGGGGGAGGGAGGGTGGCCTGCGGAGGTCCCTGGGCTGA  (4)
                       GGGGGGAGGGAGGGTGGCCTGCGGAGGTCCCTGGGCTGA  (3)
                       GGGGGGAGGGAAGGTGGCCTGCGGAGGTCCCTGGGCTGA  (2)
                        GGGGGAGGGAGGGTGGCCTGCAGACGTCCCT-GGCTGA  (1)
```

P17

```
GGGGGGAGGGAGGGAGGGAGGGAGGGAGGGTGGAGGGTGGCCTGCCGAGGTCCCTGGGCT-A  (42)
                          AGAGGGGAGAGTGGCCTGCCGAGGTCCCTGGGCTGA  (41)
                       GGGGGGAGGGAGAGTGGCCTGCGGAGGTCCCTGGGCTGA  (40)
                       GGGGGGAGGGAGGGTGGCCTGCGGAGGTCCCTGGGCTGA  (39)
                   GGGGGGAGGGAGGGAGGGTGGCCTGCGGAGGTCCCTGGGCTGA  (38)
                      GGGGGGAGGGGAGAGTGGCCTGCAGAGGTCCCTGGGCTGA  (37)
                       GGGGGGAGGGAGAGTGGCCTGCAGACATCCCTGGGCTGA  (36)
                   GGGAGGAGGGAGGGAGGGTGGCCTGCAGAGGTCCCTGGGCTGA  (35-33)
                       GGGGGGAGGGAGGGAGGCCTGCGGAGGTCCCTGGGCTGA  (32)
                       GGGGGGAGGGAGGGTGGCCTGCCGAGGTCCCTGGGCTGA  (31-41)
                       GGGGGGAGGGAGAGTGGCCTGCGGAGGTCCCTGGGCTGA  (40)
                       GGGGGGAGGGAGGGTGGCCTGCGGAGGTCCCTGGGCTGA  (39)
                   GGGGGGAGGGAGGGAGGGTGGCCTGCGGAGGTCCCTGGGCTGA  (38-5)
                       GGGGGGAGGGAGGGTGGCCTGCGGAGGTCCCTGGGCTGA  (4)
                       GGGGGGAGGGAGGGTGGCCTGCGGAGGTCCCTGGGCTGA  (3)
                       GGGGGGAGGGAAGGTGGCCTGCGGAGGTCCCTGGGCTGA  (2)
                        GGGGGAGGGAGGGTGGCCTGCAGACGTCCCT-GGCTGA  (1)
```

P21

```
GGGGGGAGGGAGGGAGGGAGGGAGGGAGGGAGGGAGGGTGGCCTGCCGAGGTCCCTGGGCTGA (42-31)
                        GGGGGGAGGGAGAGTGGCCTGCAGACATCCCTGGGCTGA (30)
                    GGGAGGAGGGAGGGAGGGTGGCCTGCAGAGGTTCCTGGGTTGA (29)
                        GGGGGGAGGGAGGGTGGCCTGCAGAGGTCCCTGGGCTGA (28)
                        GGGGGGAGGGAGGGTGGCCTGCGGAGGTCCCTGGGCTGA (27)
                        GGGGGGAGGGAGGGAGGCCTGCGGAGGTCCCTGGGCTGA (26)
                       GGGGGGAGGGGAGAGTGGCCTGCGGAGGTCCCTGGGCTGA (25-18)
                    GGGGGGAGGGAGGGAGGGTGGCCTGCGGAGGTCCCTGGGCTGA (17)
                    GGGGGGAGGGAGGGAGGGTGGCCTGCGGAGGTCCCTGGGTTGA (16-11)
                         GGGGGAGGGAGGGTGGCCTGCAGAGGTCCCTGGGCTGA (10)
                        GGAGGGAGGGAGGGTGGCCTGCGGAGGTCCCTGGGTTGA (9-11)
                         GGGGGAGGGAGGGTGGCCTGCAGAGGTCCCTGGGCTGA (10)
                        GGAGGGAGGGAGGGTGGCCTGCGGAGGTCCCTGGGCTGA (9)
                    GGGGGGAGGGAGGGAGGGTGGCCTGCCGAGGTCCCTGGGCTGA (8)
                        GGGGGGAGGGAGGGTGGCCTGCCGAGGTCCCTGGGCTGA (7)
                    GGGAGGAGGGAGGGAGGGTGGCCTGCGGAGGTCCCTGGGCTGA (6)
                        GGGGGGAGGGAGGGTGGCCTGCGGAGGTCCCTGGGCTGA (5)
                        GGGGGGAGGGAGGGTGGCCTGCGGAGGTCCCTGGGCTGA (4)
                        GGGGGGAGGGAGGGTGGCCTGCGGAGGTCCCTGGGCTGA (3)
                        GGGGGGAGGGAAGGTGGCCTGCGGAGGTCCCTGGGCTGA (2)
                         GGGGGAGGGAGGGTGGCCTGCAGACGTCCCT-GGCTGA (1)
```

P23

```
                        GGAGGGAGGGAGGGTGGCCTGCAGAGGTCCCTGGGCTGA (X-14)
                    GGGGGGAGGGAGGGAGGGTGGCCTGCGGAGGTCTCTGGGCTGA (13)
                    GGGGGGAGGGAGGGAAGGTGGCCTGCGGAGGTCCCTGGGCTGA (12)
                        GGGGGGAGGGAGGGTGGCCTGCGGAGGTCCCTGGGTTGA (11)
                         GGGGGAGGGAGGGTGGCCTGCAGAGGTCCCTGGGCTGA (10)
                        GGAGGGAGGGAGGGTGGCCTGCGGAGGTCCCTGGGCTGA (9)
                    GGGGGGAGGGAGGGAGGGTGGCCTGCCGAGGTCCCTGGGCTGA (8)
                        GGGGGGAGGGAGGGTGGCCTGCCGAGGTCCCTGGGCTGA (7)
                        GGGAGGAGGGAGGGTGGCCTGCGGAGGTCCCTGGGCTGA (6-5)
                        GGGGGGAGGGAGGGTGGCCTGCGGAGGTCCCTGGGCTGA (4-8)
                        GGGGGGAGGGAGGGTGGCCTGCCGAGGTCTCTGGGCTGA (7-13)
                    GGGGGGAGGGAGGGAAGGTGGCCTGCGGAGGTCCCTGGGCTGA (12)
                        GGGGGGAGGGAGGGTGGCCTGCGGAGGTCCCTGGGTTGA (11)
                         GGGGGAGGGAGGGTGGCCTGCAGAGGTCCCTGGGCTGA (10)
                        GGAGGGAGGGAGGGTGGCCTGCGGAGGTCCCTGGGCTGA (9-6)
                        GGGGGGAGGGAGGGTGGCCTGCGGAGGTCCCTGGGCTGA (5-8)
                        GGGGGGAGGGAGGGTGGCCTGCCGAGGTCCCTGGGCTGA (7)
                    GGGAGGAGGGAGGGAGGGTGGCCTGCGGAGGTCCCTGGGCTGA (6)
                        GGGGGGAGGGAGGGTGGCCTGCGGAGGTCCCTGGGCTGA (5)
                        GGGGGGAGGGAGGGTGGCCTGCGGAGGTCCCTGGGCTGA (4)
                        GGGGGGAGGGAGGGTGGCCTGCGGAGGTCCCTGGGCTGA (3)
                        GGGGGGAGGGAAGGTGGCCTGCGGAGGTCCCTGGGCTGA (2)
                         GGGGGAGGGAGGGTGGCCTGCAGACGTCCCT-GGCTGA (1)
```
