# Supplementary figures and images for: The Yeast Pif1 Helicase Prevents Genomic Instability Caused by G-Quadruplex-Forming CEB1 Sequences In Vivo
Source: PLoS Genet. 2009 May 8;5(5):e1000475. doi: 10.1371/journal.pgen.1000475 (PMC2673046; doi:10.1371/journal.pgen.1000475)

**Figure S2**

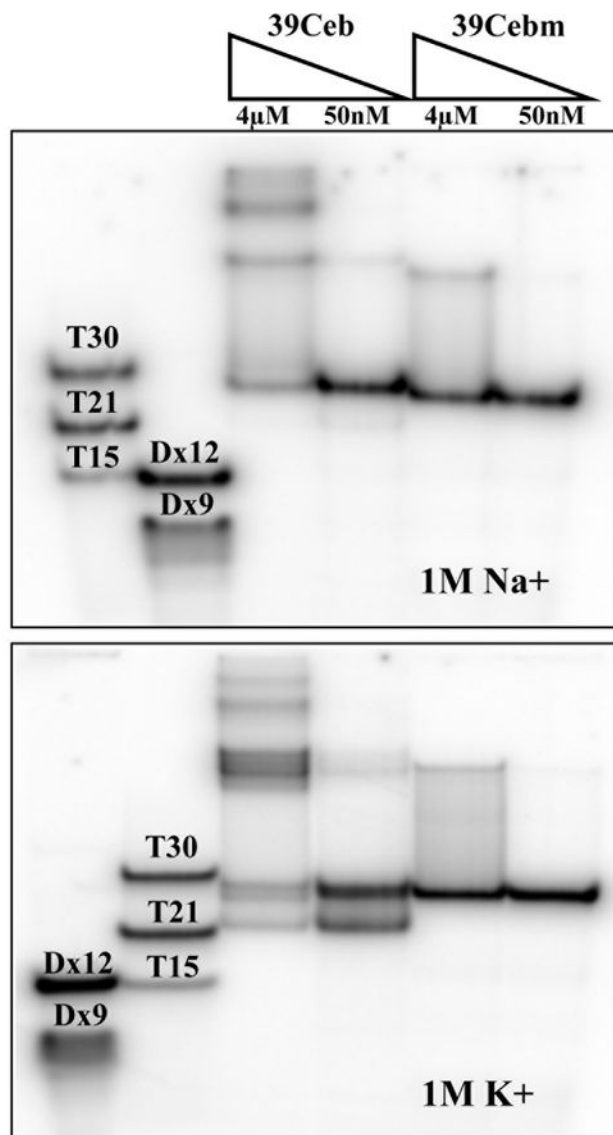

Supplement: Figure S2 — Behavior of the 39Ceb and 39Cebm sequences on a non-denaturing gel. Two strand concentrations were tested: radiolabeled only (around 50 nM) or supplemented with 4 µM of cold oligonucleotide. Samples were treated with 50 mM LiOH to unfold quadruplexes, reannealed in 1 M NaCl buffer (top) or KCl (bottom) for 2 hours and loaded on a non-denaturing 15% acrylamide gel and run at 26°C. Migration markers are double-stranded DNA (9 and 12 bp) and (dT)15, (dT)21 and (dT)30 oligomers. (0.11 MB PDF) [file pgen.1000475.s002.pdf]
