# Supplementary material for: The Yeast Pif1 Helicase Prevents Genomic Instability Caused by G-Quadruplex-Forming CEB1 Sequences In Vivo
Source: PLoS Genet. 2009 May 8;5(5):e1000475. doi: 10.1371/journal.pgen.1000475 (PMC2673046; doi:10.1371/journal.pgen.1000475)
Supplement: Figure S3 — Synthesis of artificial CEB1 minisatellites by PCR. (A) Nucleotide sequence of the CEB1-WT and CEB1-Gmut motifs. Repeats of at least three consecutive guanines are highlighted in grey in the CEB1-WT motif. Point mutations interrupting the G-triplets in the CEB1-Gmut motif are underlined. (B) Schematic representation of CEB1-concatemers synthesized by PCR. Two complementary oligonucleotides for CEB1-Gmut are represented (up and low), each composed of two identical CEB1-Gmut motifs (see Text S1 for sequences). After the first cycle of denaturation and annealing, the oligonucleotides can perfectly anneal along the two motifs and no elongation is possible (left), or they can shift and only one motif is annealed and the second motif is used as DNA template for elongation (right) resulting in addition of one motif at the end of the cycle. (C) After 30 cycles, DNA is deposited in agarose gel and the smear corresponds to a population of CEB1-concatemers of various sizes. White square indicates the part of the gel that will be cut in order to extract DNA and clone it in pGEM-T Easy vector. Sequences of the synthetic minisatellites, CEB1-WT-1.0 (D) and CEB1-Gmut-1.7 (E), with 26 and 42 repeats respectively. The sequence of the parental motif (CEB1-WT or CEB1-Gmut) used for the synthesis is indicated above the sequence of the synthetic minisatellite. Mutations and small deletions introduced during the concatemer synthesis are highlighted in red and in grey, respectively. (0.51 MB PDF) [file pgen.1000475.s003.pdf]

Figure S3

A

Motif CEB1-WT 5′ GGGGGGAGGGAGGGTGGCCTGCGGAGGTCCCTGGGCTGA 3′

Motif CEB1-Gmut 5′ GCGCGGAGTGAGAGTGGCCTGCGGAGGTCCCTGCGCTGA 3′

B

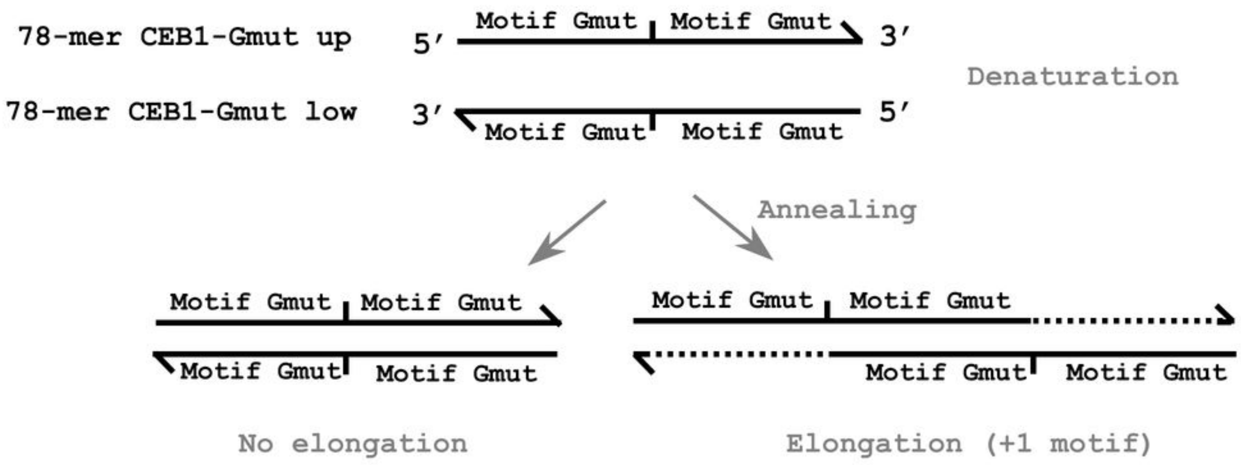

C

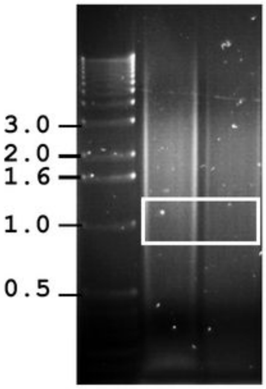

D

Synthetic CEB1-WT-1.0

Motif CEB1-WT:

5′ GGGGGGAGGGAGGGTGGCCTGCGGAGGTCCCTGGGCTGA 3′

GGGGGGAGGGAGGGTGGCCTGCGGAGGTCCCTGGGCTGA	1
GGGGGGAGGGAGGGTGGCCTGCGGAGGTCCCTGGGCTGA	2
GGGGGGAGGGAGGGTGGCCTGCGGAGGTCCCTGGGCTGA	3
GGGGGGAGGGAGGGTGGCCTGCGGAGGTCCCTGGGCTGA	4
GGGGGGAGGGAGGGTGGCCTGCGGAGGTCCCTGGGCTGA	5
GGGGGGAGGGAGGGTGGCCTGCGGAGGTCC--GGGCTGA	6
GGGGGGAGGGAGGGTGGCCTGCGGAGGTCCCTGGGCTGA	7
GGGGGGAGGGAGGGTGGCCTGCGGAGGTCCCTGGGCTGA	8
GGGGGGAGGGAGGGTGGCCTGCGGAGGTCCCTGGGCTGA	9
GGGGGGAGGGAGGGTGGCCTGCGGAGGTCCCTGGGCTGA	10
GGGGGGAGGGAGGGTGGCCTGCGGAGGTCCCTGGGCTGA	11
GGGGGGAGGGAGGGTGGCCTGCGGAGGTCCCTGGGCTGA	12
GGGGGGAGGGAGGGTGGCCTGCGGAGGTCCCTGGGCTGA	13
GGGGGGAGGGAGGGTGGCCTGCGGAGGTCCCTGGGCTGA	14
GGGGGGAGGGAGGGTGGCCTGCGGA--TCCCTGGGCTGA	15
GGGGGGAGGGAGGGTGGCCTGCGGAGGTCCCTGGGCTGA	16
GGGGGGAGGGAGGGTGGCCTGCGGAGGTCCCTGGGCTGA	17
GGGGGGAGGGAGGGTGGCCTGCGGAGGTCC-TGGGCTGA	18
GGGGGGAGGGAGGGTGGCCTGCGGAGGTCCCTGGGCTGA	19
GGGGGGAGGGAGGGTGGCCTGCGGAGGTCCCTGGGCTGA	20
GGGGGGAGGGAGGGTGGCCTGCGGAGGTCCCTGGGCTGA	21
GGGGGGAGGGAGGGTGGCCTGCGGAGGTCCCTGGGCTGA	22
GGGGGGAGGGAGGGTGGCCTGCGGAGGTCCCTGGGCTGA	23
GGGGGGAGGGAGGGTGGCCTGCGGAGGTCCCTGGGCTGA	24
GGGGGGAGGGAGGGTGGCCTGCGGAGGTCCCTGGGCTGA	25
GGGGGGAGGGAGGGTGGCCTGC	26

Figure S3

E

Synthetic CEB1-Gmut-1.7

Motif CEB1-Gmut:

```
5'GCGCGGAGTGAGAGTGGCCTGCGGAGGTCCCTGCGCTGA 3'

GCGC---------GTGGCCTGCGGAGGTCGCTGCGCTGA      1
GCGCGGAGTGAGAGTGGCCTGCGGAGGTCCCTGCGCTGA      2
GCGCGGAGTGAGAGTGGCCTGCGGAGGTCCCTGCGCTGA      3
GCGCGGAGTGAGAGTGGCCTGCGGAGGTCGCTGCGCTGA      4
GCGCGGAGTGAGAGTGGCCTGCGGAGGTCGCTGCGCTGA      5
GCGCGGAGTGAGAGTGGCCTGCGGAGGTCGCTGCGCTGA      6
GCGCGGAGTGAGAGTGGCCTGCGGAGGTCGCTGCGCTGA      7
GCGCGGAGTGAGAGTGGCCTGCGGAGGTCGCTGCGCTGA      8
GCGCGGAGTGAGAGTGGCCTGCGGAGGTCGCTGCGCTGA      9
GCGCGGAGTGAGAGTGGCCTGCGGAGGTCCCTGCGCTGA      10
GCGCGGAGTGAGAGTGGCCTGCGGAGGTCGCTGCGCTGA      11
GCGCGGAGTGAGAGTGGCCTGCGGAGGTCCCTGCGCTGA      12
GCGCGGAGTGAGAGTGGCCTGCGGAGGTCGCTGCGCTGA      13
GCGCGGAGTGAGAGTGGCCTGCGGAGGTCCCTGCGCTGA      14
GCGCGGAGTGAGAGTGG---GCGGAG--CCCTGCGCTGA      15
GCGCGGAGTGAGAGTGGCCTGCGGAGGTCGCTGCGCTGA      16
GCGCGGAGTGAGAGTGGCCTGCGGAGGTCCCTGCGCTGA      17
GCGCGGAGTGAGAGTGGCCTGCGGAGGTCCCTGCGCTGA      18
GCGCGGAGTGAGAGTGGCCTGCGGAGGTCCCTGCGCTGA      19
GCGCGGAGTGAGAGTGGCCTGCGGAGGTCGCTGCGCTGA      20
GCGC-GAGTGAGAGTGGCCTGCGGAGGTCGCTGCGCTGA      21
GCGCGGAGTGAGAGTGGCCTGCGGAGGTCGCTGCGCTGA      22
GCGCGGAGTGAGAGTGGCCTGCGGAGGTCGCTGCGCTGA      23
GCGCGGAGTGAGAGTGGCCTGCGGAGGTCGCTGCGCTGA      24
GCGCG-AGTGAGAGTGGCCTGCGGAGGTCGCTGCGCTGA      25
GCGCGGAGTGAGAGTGGCCTGCGGAGGTCGCTGCGCTGA      26
GCGCGGAGTGAGAGTGGCCTGCGGAGGTCGCTGCGCTGA      27
GCGCGGAGTGAGAGTGGCCTGCGGAGGTCCCTGCGCTGA      28
GCGCGGAGTGAGAGTGGCCTGCGGAGGTCGCTGCGCTGA      29
GCGCGGAGTGAGAGTGGCCTGCGGAGGTCCCTGCGCTGA      30
GCGCGGAGTGAGAGTGGCCTGCGGAGGTCGCTGCGCTGA      31
GCGCGGAGTGAGAGTGGCCTGCGGAGGTCGCTGCGCTGA      32
GCGCGGAGTGAGAGTGGCCTGCGGAGGTCCC-GCG-TGA      33
GCGCGGAGTGAGAGTGGCCTGCGGAGGTCGCTGCGCTGA      34
GCGCGGAGTGAGAGTGGCCTGCGGAGGTCGCTGCGCTGA      35
GCGCGGAGTGAGAGTGGCCTGCGGAGGTCGCTGCGCTGA      36
GCGCGGAGTGAGAGTGGCCTGCGGAGGTCGCTGCGCTGA      37
GCGCGGAGTGAGAGTGGCCTGCGGAGGTCCCTGCGCTGA      38
GCGCGGAGTGAGA-TGGCCTGCGGAGGTCGCTGCGCTGA      39
GCGCGGAGTGAGAGTGGCCTGCGGAGGTCGCTGCG-TGA      40
GCGCGGAGTGAGAGTGGCCTG-------CCCTGCGCTGA      41
GCGCGGAGTGAGAGTGGCCTGCGGAGGTCCCTGCGCTGA      42
```
