# Supplementary material for: The Yeast Pif1 Helicase Prevents Genomic Instability Caused by G-Quadruplex-Forming CEB1 Sequences In Vivo
Source: PLoS Genet. 2009 May 8;5(5):e1000475. doi: 10.1371/journal.pgen.1000475 (PMC2673046; doi:10.1371/journal.pgen.1000475)
Supplement: Table S1 — List of strains used in this study. (0.10 MB PDF) [file pgen.1000475.s004.pdf]

TABLE S1. Genotypes of yeast strains used in this study

Strain	Relevant genotype	Source
ORT2914	*MAT α* CEB1-1.8 *ARG4*	Lopes *et al.* 2006
ORT4840	*MAT α srs2:: KanMX4* CEB1-1.8 *ARG4*	This study
ORT4849	*MAT α sgs1:: KanMX4* CEB1-1.8 *ARG4*	This study
ORT4880	*MAT α rrm3:: KanMX4* CEB1-1.8 *ARG4*	This study
ORT4885	*MAT α mph1:: KanMX4* CEB1-1.8 *ARG4*	This study
ORT5084-2C	*MAT α̣ pif1-m1* CEB1-1.8 *ARG4*	This study
ORT5085-1C	*MAT α̣ pif1-m2* CEB1-1.8 *ARG4*	This study
ORT5083-4E	*MAT α̣ pif1-K264R* CEB1-1.8 *ARG4*	This study
ORT5087-5E	*MAT α̣ pif1-K264A* CEB1-1.8 *ARG4*	This study
ORT4841	*MAT α pif1:: KanMX4* CEB1-1.8 *ARG4*	This study
ORT4843	*MAT α pif1:: KanMX4* CEB1-1.8 *ARG4*	This study
ORD7557-10B	*MAṬ α pif1:: KanMX4* CEB1-0.6 *ARG4*	This study
ORD7598-12C	*MAT α pif1:: KanMX4* CEB1-3.0 *ARG4*	This study
ORT4841-4E1	*MAT α pif1:: KanMX4* CEB1-3.5 *ARG4*	This study
ORD7569	*MAT a/α pif1:: KanMX4/pif1:: KanMX4* CEB1-1.8 *ARG4*/ CEB1-1.8 *ARG4*	This study
ORT5086	*MAT α pif1:: KanMX4* CEB1-1.8 *ARG4* + pJL76	This study
ORT4848	*MAT α pif1:: KanMX4 dna2::HygMX4* CEB1-1.8 *ARG4*	This study
ORT5604	*MAT α* CEB1-1.8 *ARG4* + pMD28	This study
ORT5606	*MAT α* CEB1-1.8 *ARG4* + pBK1	This study
ORT5614	*MAT α* CEB1-1.8 *ARG4* + pBK3	This study
ORT5602	*MAT α* CEB1-1.8 *ARG4* + pBK10	This study
ORT5608	*MAT α* CEB1-1.8 *ARG4* + pEAS20	This study
ORT5600	*MAT α pif1:: KanMX4* CEB1-1.8 *ARG4* + pMD28	This study
ORT4896	*MAT α pif1:: KanMX4* CEB1-1.8 *ARG4* + pBK1	This study
ORT4894	*MAT α pif1:: KanMX4* CEB1-1.8 *ARG4* + pBK3	This study
ORT4892	*MAT α pif1:: KanMX4* CEB1-1.8 *ARG4* + pBK10	This study
ORT4898	*MAT α pif1:: KanMX4* CEB1-1.8 *ARG4* + pEAS20	This study
AND1228-2A	*MAT a pif1:: KanMX4* hRAS1-2.1 *ARG4*	This study
AND1228-8C	*MAT α rad27::HIS3* hRAS1-2.1 *ARG4*	This study
ORT6009	*MAT α rad27::HIS3* CEB1-1.8 *ARG4* + pMD28	This study
ORT6014	*MAT α rad27::HIS3* CEB1-1.8 *ARG4* + pBK1	This study
ORT6013	*MAT α rad27::HIS3* CEB1-1.8 *ARG4* + pBK3	This study
ORT6011	*MAT α rad27::HIS3* CEB1-1.8 *ARG4* + pBK10	This study
ORT6016	*MAT α rad27::HIS3* CEB1-1.8 *ARG4* + pEAS20	This study
ORD7574-11C	*MAT α rad51::LEU2* CEB1-1.8 *ARG4*	This study
ORD6786-4A	*MAT α rif1::KanMX4* CEB1-1.8 *ARG4*	This study
ORD6713-8D	*MAT a rad27::HIS3* CEB1-1.8 *ARG4*	Lopes *et al.* 2006
ORD7574-9B	*MAT α pif1:: KanMX4 rad51::LEU2* CEB1-1.8 *ARG4*	This study
ORD7565-2C	*MAT a pif1:: KanMX4 rad52::LEU2* CEB1-1.8 *ARG4*	This study
ORD9922-4B	*MAT α pif1:: HIS3 sgs1::KanMX4* CEB1-1.8 *ARG4*	This study
ORD9304-9A	*MAT α pif1:: HIS3 rrm3::KanMX4* CEB1-1.8 *ARG4*	This study
ORD6708	*MAT a/α rad27::HIS3/ rad27::HIS3 CEB1-1.8 ARG4/ CEB1-0.6 arg4bg*	Lopes *et al.* 2002
AND1207-9B	*MAT α* CEB1-WT-2.3 *ARG4*	This study
AND1212-10D	*MAT a* CEB1-WT-1.7 *ARG4*	This study
AND1213-1D	*MAT a* CEB1-WT-1.0 *ARG4*	This study
AND1202-13D-P14C3	*MAT α pif1:: KanMX4* CEB1-WT-1.9 *ARG4*	This study
AND1202-11A	*MAT a pif1:: KanMX*4 CEB1-WT-1.7 *ARG4*	This study
AND1202-11A-L8C12	*MAT a pif1:: KanMX4* CEB1-WT-1.3 *ARG4*	This study
ORT6108-4	*MAT α pif1:: KanMX4* CEB1-WT-1.0 *ARG4*	This study
AND1218-1A	*MAT α rad27::HIS3* CEB1-WT-1.7 *ARG4*	This study
ORT6110-1	*MAṬ α rad27::HIS3* CEB1-WT-1.0 *ARG4*	This study
AND1206-5D	*MAT a* CEB1-Gmut-3.8 *ARG4*	This study
AND1227-5C	*MAT α* CEB1-Gmut-1.7 *ARG4*	This study
AND1206-4C	*MAT a pif1:: KanMX4* CEB1-Gmut-3.8 *ARG4*	This study
AND1206-4C-1B6-1E1	*MAT a pif1:: KanMX4* CEB1-Gmut-2.5 *ARG4*	This study
AND1206-4C-D11P2	*MAT a pif1:: KanMX4* CEB1-Gmut-1.7 *ARG4*	This study
ORT6107-1	*MAT α pif1:: KanMX4* CEB1-Gmut-0.7 *ARG4*	This study
AND1206-4B	*MAT α rad27::HIS3* CEB1-Gmut-3.8 *ARG4*	This study
AND1226-18B/-17C	*MAT α rad27::HIS3* CEB1-Gmut-1.7 *ARG4*	This study
ORT6109	*MAT α rad27::HIS3* CEB1-Gmut-0.7 *ARG4*	This study
