# Supplementary material for: The Yeast Pif1 Helicase Prevents Genomic Instability Caused by G-Quadruplex-Forming CEB1 Sequences In Vivo
Source: PLoS Genet. 2009 May 8;5(5):e1000475. doi: 10.1371/journal.pgen.1000475 (PMC2673046; doi:10.1371/journal.pgen.1000475)
Supplement: Table S2 — Sequence of the oligonucleotides used and their respective melting temperatures. (0.08 MB PDF) [file pgen.1000475.s005.pdf]

Table S2 : Sequence of the oligonucleotides used and their respective melting temperatures

**Name** [a]	**Sequence (5’ => 3’)** [b]	**Tm** [c] **Na^+ (°C)**	**Tm** [c] **K^+ (°C)**
24Ceb	TGAGGGGGGAGGGAGGGAGGGTGG	**42**	**69**
29Ceb	AGGGGGGAGGGAGGGTGGCCTGCGGAGGT	**38**	**54**
39Ceb	AGGGGGGAGGGAGGGTGGCCTGCGGAGGTCCCTGGGCTG	**48**	**55**
24Cebm	TGAG**C**G**C**GGAG**T**GAG**A**GTGGCCTG	*dx*	*dx*
39Cebm	AG**C**G**C**GGAG**T**GAG**A**GTGGCCTGCGGAGGTCCCTG**C**GCTG	*dx*	*dx*
28hRAS1	GGCGTCCCCTGGACAGAAGGGGGAGTGT	*dx*	*dx*
28hRAS2	GGCATTCCCTGGACAGAAGGGCAAGTGT	*dx*	*dx*
28hRAS3	GGCGTCCCCTGGAGAGAAGGGCCAGTGT	*dx*	*dx*

[a] 24Ceb, 29Ceb and 39Ceb oligonucleotides correspond to sequences found in CEB1 minisatellite repeats. 24Cebm and 39Cebm are mutant sequences in which guanines blocks have been disrupted. 28hRAS1, 28hRAS2 and 28hRAS3 are control sequences found in *hRAS1* GC rich minisatellite. [b] Blocks of 3 or more guanines are shown in red. Mutations that interrupt guanine blocks appear in bold/underlined. [c] Melting temperature, in °C (average of 2-4 independent experiments) determined in a 10 mM lithium pH 7.2 cacodylate buffer supplemented with 0.1M NaCl or KCl (last column). Values in bold were determined at 295 nm and could be attributed to quadruplex thermal denaturation. *dx*: no transition at 295 nm but evidence for duplex formation, with a melting temperature >37°C.
