# Supplementary material for: The Yeast Pif1 Helicase Prevents Genomic Instability Caused by G-Quadruplex-Forming CEB1 Sequences In Vivo
Source: PLoS Genet. 2009 May 8;5(5):e1000475. doi: 10.1371/journal.pgen.1000475 (PMC2673046; doi:10.1371/journal.pgen.1000475)
Supplement: Text S1 — Supplementary material and methods. (0.13 MB PDF) [file pgen.1000475.s006.pdf]

SUPPLEMENTAL DATA

Supplemental Materials and Methods

Yeast strains and plasmids. *S. cerevisiae* strains were grown as previously described [1]. The relevant genotypes and sources of haploid and diploid *S. cerevisiae* strains (S288C background) used in this study are indicated in Table S1. The CEB1-0.6, CEB1-1.8 and CEB1-3.0 alleles were inserted on chromosome VIII in the *ARG4* promoter as described previously [1,2]. The CEB1-3.5 allele is an expansion of CEB1-1.8 isolated from ORT4841.

The *KanMX4* disrupted strains were constructed by PCR amplification of the *geneX*::*KanMX4* cassette with flanking regions from the BY strains of the EUROSCARF deletants collection. After transformation of the yeast strain ORT2914 with the purified PCR product, $G418^R$ strains were verified for correct chromosomal integration by Southern blot. *pif1*::*HIS3* strains were built using plasmid M4754 (provided by David Stillman) which allows replacement of *KanMX4* by *HIS3* [3]. The *rad51::LEU2* and the *rad52::LEU2* disruptions originated from ORT2615 and ORT2916 respectively [4] were introduced by genetic crosses. To construct *DNA2* deletion, the *HygMX4* cassette was amplified from pAG32 [5] with primers containing homology with flanking regions of *DNA2:* DNA2ApMJ696A (5'-GAGTACTCATTTGTGCAAGCAAACACTGACAATTGAAGAGATCGTCAGGATCCCCGGGTTAATTAAGGC-3') and DNA2BpMJ696B (5'-AGCTCGTTTTCGACACTGGTGCTGTGATAGCTTTCCTGTTATGGAGAAGCTCTTCTTATTCCCCCTGTC-3'). The PCR product was used to transform ORT4841 resulting in ORT4848.

The strains ORT4841, ORT2914 and ORD6713-8D were all transformed with replicative plasmids pBK3, pBK1, pMD28, pEAS20 and pBK10 [6]. These plasmids contain various

repetitive tracts in frame with the *URA3* marker for examining micro- and minisatellites instability.

The *hRAS1* minisatellite was amplified by PCR from the p37Y8 plasmid (gift from D. Kirkpatrick) using Ras 5’ and Ras 3’ primers and Taq polymerase (Invitrogen) as previously described [7]. The PCR fragment was cloned in pGEM-T Easy vector (Promega) and the *Apa*I-*Spe*I fragment containing *hRAS1* was inserted in pJL82 plasmid derived from pUC19. pJL82 contains a 3.1 kb *Hind*III-*Pst*I fragment from *DED81-ARG4* region in which an unrelated polylinker DNA sequence, polyI, replaces a part of *ARG4* promoter [2]. *arg-* strains (WT, *pif1*Δ and *rad27*Δ) were transformed by electroporation with *Hind*III-*Pst*I fragment containing minisatellite and selected for arginine prototrophy. Correct integration was verified by Southern blot analysis of Arg^+ transformants using *EcoR*V/*Pvu*II digestion and a *DED81* probe.

In order to create *PIF1* point mutations, a *PIF1* fragment was amplified from yeast genomic DNA using PIF1-223-XhoI (5’-TCATGCTCGAGACATTAAGAAAGGCGCGTCT-3’) and PIF1+1023-XbaI (5’-GAATCTCTAGAATCGACAACCAAAGCACCAA-3’) primers. This fragment was digested by *Xba*I/*Xho*I (highlighted in primers) and inserted into integrative vector pRS306 containing *URA3* marker at *Xba*I/*Xho*I sites to create pJL69. The pJL69 vector was next used to create *PIF1* point mutations by site directed mutagenesis using QuikChange Site-Directed Mutagenesis (Stratagene). The following primers were used (mutated bases are highlighted): *pif1-K264R* was created using PIF1-K264R-up (5’-GAGTGCCGGTACCGGTAGATCCATTCTTTTACGTG-3’) and PIF1-K264R-low (5’-CACGTAAAAGAATGGATCTACCGGTACCGGCACTC-3’), *pif1-m1* using PIF1-M1-up (5’-CTTGTATCAATCAATTTTGGGCCCCAAAGTGGATAAGATC-3’) and PIF1-M1-low (5’-GATCTTATCCACTTTGGGGCCCAAAATTGATTGATACAAG-3’), *pif1-m2* using PIF1-M2-up (5’-CCAAACTATCGTTTTCTGCCTCGAGTCGTGGTTTCAGGTC-3’) and PIF1-M2-low (5’-

GACCTGAAACCACGACTCGAGGCAGAAAACGATAGTTTGG-3'). After verification by sequencing of the presence of the mutation, a *Xba*I/*Xho*I fragment (*pif1-K264R* and *pif1-m1*) or a *Xba*I/*Bse*RI fragment (*pif1-m2*) was cloned into pRS306 plasmid at the same sites (*Xba*I/*Xho*I for *pif1-K264R* and *pif1-m1; Xba*I/*Bse*RI for *pif1-m2*). This creates pJL71 (*pif1-K264R*), pJL72 (*pif1-m1*) and pJL73 (*pif1-m2*).

For *pif1-m2* overexpression, the entire ORF containing *pif1-m2* mutation plus the 5' and 3' noncoding region was amplified from yeast genomic DNA (from strain ORT5085-1C) with primers PIF1A (5'-AATGGCAAGTTTGCCGAA-3') and PIF1B (5'-TGCTTCCTGTCAGCTTGGTT-3'); next this PCR product was cloned in pCR2.1 vector (Invitrogen) which creates pJL75. A *Sac*I/*Not*I fragment from pJL75 containing the entire PCR product was introduced in a 2µ replicative plasmid pRS426 containing the *URA3* marker to create pJL76. This plasmid was transformed in the yeast strain ORT4841 and the resulting transformants (ORT5086) were selected for uracil prototrophy.

The *Pfl*MI/*Bse*RI fragment from pVS102PKA [8], which carries *pif1-K264A* mutation, was inserted at *Pfl*MI/*Bse*RI sites in pJL75, replacing *pif1-m2* mutation by *pif1-K264A* mutation, to create pJL78. The *Sac*I/*Not*I fragment, containing the entire ORF with *pif1-K264A* mutation plus the 5' and 3' noncoding region from pJL78 was inserted at *Sac*I/*Not*I in pRS306 plasmid, which creates pJL79. The plasmids pJL71 (*pif1-K264R*), pJL72 (*pif1-m1*), pJL73 (*pif1-m2*) and pJL79 (*pif1-K264A*) were linearized with *Bsu36*I, *Afl*II, *Afl*II and *BstE*II respectively, and introduced by transformation pop-in pop-out at the *PIF1* locus in the yeast strain ORT2914. Correct integration of the plasmids was verified by Southern blot analysis.

Instability of tandem repeated sequences on plasmids. For plasmids pBK3, pBK1, pMD28, pEAS20 and pBK10, alteration in tract length results in an out of frame insertion

which can be selected on medium containing 5-Fluoro-Orotic-Acid (5-FOA) as previously described [6].

Synthetic minisatellites. The synthetic minisatellites were generated by PCR using complementary primers: 39x2-up (5' TCAGCCCAGGGACCTCCGCAGGCCACCCTCCCTCCCCCTCAGCCCAGGGACCTCCGCAGGCCACCCTCCCTCCCCC 3') and 39x2-low (5' GGGGGAGGGAGGGTGGCCTGCGGAGGTCCCTGGGCTGAGGGGGAGGGAGGGTGGCCTGCGGAGGTCCCTGGGCTGA 3') for the CEB1-WT minisatellites and the primers 39x2mut-up (5' TCAGCGCAGGGACCTCCGCAGGCCACTCTCACTCCGCGCTCAGCGCAGGGACCTCCGCAGGCCACTCTCACTCCGCGC 3') and 39x2mut-low (5' GCGCGGAGTGAGAGTGGCCTGCGGAGGTCCCTGCGCTGAGCGCGGAGTGAGAGTGGCCTGCGGAGGTCGCTGCGCTGA 3') for the CEB1-Gmut minisatellites. Mutations interrupting each triplet of guanines are underlined. Principle of the minisatellite synthesis is represented in Figure S3 B. PCR reaction was done with a final concentration of 0.37 μM for each oligonucleotide, the PFU Ultra polymerase (1U) (Stratagene) in a total volume of 15 μl using the buffer described in Jeffreys *et al.* [9]. Reaction conditions were: 3 min at 95°C for one cycle, and 30 s at 95°C, 30 s at 64°C and 30 s at 72°C +2 s/cycle for 30 cycles and the reaction was terminated by 10 min at 72°C. At the end of the PCR, 0.2μl of recombinant Taq polymerase (Invitrogen) was added during 6 min at 72°C for A-tailing procedure. To increase the quantity of amplified DNA, four identical PCR reactions were performed in parallel. These PCR products were pooled, precipitated and electrophoresed in 0.8% agarose gel. DNA fragments around 1 kb were extracted from agarose gel with NucleoSpin Extract II kit (Macherey–Nalgen), cloned in pGEM-T Easy vector (Promega) and sequenced with the Big Dye Terminator version 3.1 kit (Perkin Elmer) and 20% Betaine (Sigma) (Figure S3). Synthetic CEB1 minisatellites were inserted on chromosome VIII in the *ARG4* promoter as described for *hRAS1* minisatellite.

Supplemental References

1. Lopes J, Ribeyre C, Nicolas A (2006) Complex minisatellite rearrangements generated in the total or partial absence of Rad27/hFEN1 activity occur in a single generation and are Rad51 and Rad52 dependent. Mol Cell Biol 26: 6675-6689.
2. Debrauwere H, Buard J, Tessier J, Aubert D, Vergnaud G, et al. (1999) Meiotic instability of human minisatellite CEB1 in yeast requires DNA double-strand breaks. Nat Genet 23: 367-371.
3. Voth WP, Jiang YW, Stillman DJ (2003) New 'marker swap' plasmids for converting selectable markers on budding yeast gene disruptions and plasmids. Yeast 20: 985-993.
4. Soustelle C, Vedel M, Kolodner R, Nicolas A (2002) Replication protein A is required for meiotic recombination in *Saccharomyces cerevisiae*. Genetics 161: 535-547.
5. Goldstein AL, McCusker JH (1999) Three new dominant drug resistance cassettes for gene disruption in *Saccharomyces cerevisiae*. Yeast 15: 1541-1553.
6. Kokoska RJ, Stefanovic L, Tran HT, Resnick MA, Gordenin DA, et al. (1998) Destabilization of yeast micro- and minisatellite DNA sequences by mutations affecting a nuclease involved in Okazaki fragment processing (rad27) and DNA polymerase delta (pol3-t). Mol Cell Biol 18: 2779-2788.
7. Jauert PA, Edmiston SN, Conway K, Kirkpatrick DT (2002) *RAD1* controls the meiotic expansion of the human HRAS1 minisatellite in *Saccharomyces cerevisiae*. Mol Cell Biol 22: 953-964.
8. Zhou J, Monson EK, Teng S, Schulz VP, Zakian VA (2000) Pif1p helicase, a catalytic inhibitor of telomerase in yeast. Science 289: 771-774.
9. Jeffreys AJ, Neumann R, Wilson V (1990) Repeat unit sequence variation in minisatellites: a novel source of DNA polymorphism for studying variation and mutation by single molecule analysis. Cell 60: 473-485.
